# C3G promotes a selective release of angiogenic factors from activated mouse platelets to regulate angiogenesis and tumor metastasis

**DOI:** 10.18632/oncotarget.22339

**Published:** 2017-11-06

**Authors:** Víctor Martín-Granado, Sara Ortiz-Rivero, Rita Carmona, Sara Gutiérrez-Herrero, Mario Barrera, Laura San-Segundo, Celia Sequera, Pedro Perdiguero, Francisco Lozano, Francisco Martín-Herrero, José Ramón González-Porras, Ramón Muñoz-Chápuli, Almudena Porras, Carmen Guerrero

**Affiliations:** ^1^ Instituto de Biología Molecular y Celular del Cáncer, USAL-CSIC, Salamanca, Spain; ^2^ Instituto de Investigación Biomédica de Salamanca (IBSAL), Salamanca, Spain; ^3^ Departamento de Biología Animal, Universidad de Málaga, Málaga, Spain; ^4^ Departamento de Bioquímica y Biología Molecular II, Facultad de Farmacia, Universidad Complutense de Madrid, Instituto de Investigación Sanitaria del Hospital Clínico San Carlos (IdISSC), Madrid, Spain; ^5^ Departamento de Cardiología, Hospital Universitario de Salamanca, Universidad de Salamanca, Salamanca, Spain; ^6^ Departamento de Angiología y Cirugía Vascular, Hospital Universitario de Salamanca, Universidad de Salamanca, Salamanca, Spain; ^7^ Departamento de Hematología, Hospital Universitario de Salamanca, Universidad de Salamanca, Salamanca, Spain; ^8^ Departamento de Medicina, Universidad de Salamanca, Salamanca, Spain

**Keywords:** C3G, platelet secretome, angiogenesis, Vamp-7, metastasis

## Abstract

Previous observations indicated that C3G (RAPGEF1) promotes α-granule release, evidenced by the increase in P-selectin exposure on the platelet surface following its activation. The goal of the present study is to further characterize the potential function of C3G as a modulator of the platelet releasate and its implication in the regulation of angiogenesis.

Proteomic analysis revealed a decreased secretion of anti-angiogenic factors from activated transgenic C3G and C3G∆Cat platelets. Accordingly, the secretome from both transgenic platelets had an overall pro-angiogenic effect as evidenced by an *in vitro* capillary-tube formation assay with HUVECs (human umbilical vein endothelial cells) and by two *in vivo* models of heterotopic tumor growth. In addition, transgenic C3G expression in platelets greatly increased mouse melanoma cells metastasis. Moreover, immunofluorescence microscopy showed that the pro-angiogenic factors VEGF and bFGF were partially retained into α-granules in thrombin- and ADP-activated mouse platelets from both, C3G and C3GΔCat transgenic mice. The observed interaction between C3G and Vesicle-associated membrane protein (Vamp)-7 could explain these results. Concomitantly, increased platelet spreading in both transgenic platelets upon thrombin activation supports this novel function of C3G in α-granule exocytosis.

Collectively, our data point out to the co-existence of Rap1GEF-dependent and independent mechanisms mediating C3G effects on platelet secretion, which regulates pathological angiogenesis in tumors and other contexts. The results herein support an important role for platelet C3G in angiogenesis and metastasis.

## INTRODUCTION

C3G, also known as RAPGEF1, is a guanine nucleotide exchange factor (GEF) for Rap1 and R-Ras proteins that participates in numerous cellular processes such as proliferation, differentiation, migration, apoptosis and cell-cell contact maintenance [1–8]. C3G is a multidomain protein consisting of a N-Terminal domain, which interacts with E-cadherin, a central region with several polyproline tracts (SH3-binding domain) capable of interacting with proteins containing SH3 domains, and a carboxy-terminal region that includes the catalytic (REM-CDC25) domain, responsible for its GEF activity [6]. Many C3G functions are mediated by interaction with other proteins through its SH3-binding domain, independently of its catalytic (GEF) activity [1, 3–8].

Using transgenic mouse models expressing human full-length C3G or mutant C3G∆Cat (with a deletion in the catalytic domain), specifically in megakaryocytes and platelets, we previously showed that C3G increased platelet activation and aggregation, both *in vitro* and *in vivo* [9]. Specifically, C3G participates in platelet functions triggered by thrombin, ADP, PMA and collagen through the activation of its main effector Rap1b, which is known to play an important role in primary hemostasis by targeting αIIbβ3 integrin [10, 11]. In particular, we demonstrated that C3G mediates the activation of Rap1 induced by thrombin and PMA via protein kinase C (PKC) [9].

In addition to their essential functions in hemostasis and thrombosis, platelets also play a role in the regulation of immune responses, cancer metastasis, vascular development and angiogenesis [12, 13]. Platelets are recruited to sites of vascular injury where they interact with the endothelium to regulate angiogenesis [14]. Platelets contribute to this process by providing both, pro-angiogenic factors and angiogenic inhibitors, which are localized in secretory α-granules [15]. There is controversy on how proteins with potentially antagonistic functions are packaged within α-granules. Some authors have described a differential segregation of pro-angiogenic and anti-angiogenic proteins into different sets of platelet α-granules, which are selectively released in response to distinct agonists [16, 17]. According to them, pro-angiogenic granules are released in response to ADP, PAR1 or GPVI, while secretion of anti-angiogenic proteins is triggered by PAR4 or TXA_2_ activation [17, 18]. However, data from other authors suggest that protein delivery to individual granules, as well as platelet secretion, are stochastic processes and that pro- and anti-angiogenic α-granule proteins show low functional co-clustering [19, 20].

Some of the mechanisms that control platelet α-granule secretion have begun to be elucidated at the molecular level. Vesicular Soluble N-ethylmaleimide-sensitive factor Attachment protein REceptors (v-SNAREs), present in α-granules, associate to target membrane SNAREs (t-SNAREs) to generate the energy required for membrane fusion [21, 22]. α-granules are heterogeneous in the content of v-SNAREs VAMPs isoforms, which correlates with functionally distinct α-granules subtypes. In spreading human platelets, only α-granules expressing VAMP-7 translocate to peripheral lamellipodia and pseudopodia, while granules containing VAMP-3 and VAMP-8 remain in the central granulomere [23]. This indicates the participation of VAMP-7, but not VAMP-3 or VAMP-8, in spreading. In addition to its SNARE domain, VAMP-7 contains an N-terminal, profilin-like longin domain that binds to G-actin and ARP2/3, suggesting its putative participation in actin remodeling [23, 24].

Angiogenesis is critical to allow tumor growth and platelets play an important role in the regulation of tumor angiogenesis [25, 26]. Therefore, based on the previously identified function of C3G in platelet activation, we hypothesized that platelet C3G could regulate tumor growth by modulating angiogenesis.

In this paper we show that C3G regulates mouse platelet secretome. Specifically, C3G controls the secretion of pro- and anti-angiogenic factors from thrombin- or ADP-stimulated mouse platelets, which results in the modulation of angiogenesis, both *in vivo* and *in vitro,* including tumor angiogenesis and metastasis. In contrast to its role in platelet aggregation, the function of C3G in platelet secretion appears to be mainly independent of its GEF activity. The interaction between C3G and Vamp-7 may explain, at least in part, this novel C3G function.

## RESULTS

### Release of anti-angiogenic factors is reduced in tgC3G and tgC3G∆Cat mouse platelets, following activation with thrombin or ADP

C3G overexpression in platelets increases P-selectin expression on the surface [9]. Since P-selectin is stored in α-granules, but it is present only in the membrane of activated platelets, we wanted to study whether C3G could regulate platelet activity by modulating the secretion of factors stored in its granules. To do this, we performed a proteomic analysis by LC-MS/MS using secretome from equal number of thrombin- or ADP-stimulated platelets of the different genotypes (tgC3G, tgC3G∆Cat and their respective wild types). The purity of the releasate fraction was confirmed by the absence of platelet membrane–specific protein αIIb (Supplementary Figure 1). Table 1 summarizes the 20 most abundant, differentially regulated, proteins released from thrombin- or ADP-activated mouse platelets. Remarkably, release of the anti-angiogenic factors thrombospondin-1 (TSP-1), platelet factor 4 (PF4) and von Willebrand factor (vWF), all of them in the top 20, was diminished in tgC3G and tgC3GΔCat mouse platelets, following activation with thrombin. Similar results were found in the ADP-induced releasates, although in this case only TSP-1 and PF4 were among the top 20 (vWF: rank 36 in tgC3G, rank 52 in tgC3G∆Cat) (Table 1 and Supplementary Table 2). Additional data containing the top 100 differentially regulated proteins per treatment are displayed in Supplementary Tables 1 and 2. Apart from TSP-1, PF4 and vWF, the release of other anti-angiogenic factors, such as α-1-antitrypsin 1-5 and Metalloproteinase inhibitor 3, was reduced in thrombin-activated tgC3G and tgC3G∆Cat platelets. In addition, α-2-macroglobulin secretion was also decreased in tgC3G platelets. Regarding secretomes from ADP-stimulated platelets, we found lower secretion of α-2-macroglobulin, α-1-antitrypsin 1-2, α-1-antitrypsin 1-4 and α-1-antitrypsin 1-5 in both transgenics.

**Table 1:** Top 20 most abundant proteins released from thrombin- or ADP-activated mouse platelets of each genotype

Top 20 proteins from thrombin-induced releasates							
Accession no.	Protein identity	MW [kDa]	#PSM				Ortholog
			2C1-	2C1+	8A3-	8A3+	
P07724	Serum albumin	68.6	603	635	650	790	^*^^†^
Q80YQ1	Thrombospondin-1	129.6	194	151	163	99	^*^^†^
Q921I1	Serotransferrin	76.6	129	111	127	158	^*^^†^
Q9Z126	Platelet factor 4	11.2	99	85	71	65	^*^^†^
Q61838	Alpha-2-macroglobulin	165.7	68	41	67	89	^*^^†^
Q00623	Apolipoprotein A-I	30.5	47	34	42	71	^*^^†^
A8DUK4	Beta-globin	15.7	37	-	54	66	^*^^†^
P60710	Actin, cytoplasmic 1	41.7	48	37	59	29	^*^^†^
P63260	Actin, cytoplasmic 2	41.7	47	36	57	29	^*^^†^
P26039	Talin-1	269.6	47	21	55	31	^*^^†^
A0A0R4J0I1	MCG1051009 (Serpin3K)	46.6	24	20	54	56	―
P01942	Hemoglobin subunit alpha	15.0	42	26	-	60	^*^^†^
E9PV24	Fibrinogen alpha chain	87.3	39	18	31	-	^*^^†^
Q91X72	Hemopexin1	51.2	28	17	25	23	^*^^†^
P21614	Vitamin D-binding protein	53.5	21	14	26	26	^*^^†^
P01027	Complement C3	186.3	25	6	28	22	^*^
E9QPU1	von Willebrand factor	308.9	25	12	29	19	^*^^†^
P20918	Plasminogen	90.7	22	2	30	22	^*^^†^
P28665	Murinoglobulin-1	165.1	14	7	26	12	―
Q00896	Alpha-1-antitrypsin 1-3	45.7	13	14	14	22	^*^^†^

Top 20 proteins from ADP-induced releasates							
Accession no.	Protein identity	MW [kDa]	#PSM				Ortholog
			2C1-	2C1+	8A3-	8A3+	
P07724	Serum albumin	68,6	501	684	616	685	^*^^†^
Q921I1	Serotransferrin	76,7	110	117	115	120	^*^^†^
Q61838	Alpha-2-macroglobulin	165,7	81	51	73	58	^*^^†^
Q80YQ1	Thrombospondin 1	129,6	72	62	84	44	^*^^†^
Q00623	Apolipoprotein A-I	30,6	29	30	47	66	^*^^†^
P60710	Actin, cytoplasmic 1	41,7	48	24	45	29	^*^^†^
A0A0R4J0I1	MCG1051009	46,6	43	20	43	39	―
P63260	Actin, cytoplasmic 2	41,8	46	22	43	26	^*^^†^
P26039	Talin-1	269,7	50	19	37	16	^*^^†^
P01027	Complement C3	186,4	42	25	23	19	^*^
Q91X72	Hemopexin	51,3	41	24	23	15	^*^^†^
P21614	Vitamin D-binding protein	53,6	33	26	22	19	^*^^†^
Q8VDD5	Myosin-9	226,2	38	17	25	7	^†^
B7FAU9	Filamin, alpha	280,3	21	18	18	13	^*^^†^
Q8K0E8	Fibrinogen beta chain	54,7	40	7	14	7	^*^^†^
P20918	Plasminogen	90,7	30	10	16	11	^*^^†^
Q00896	Alpha-1-antitrypsin 1-3	45,8	14	14	19	16	^*^^†^
P28665	Murinoglobulin-1	165,2	24	7	17	13	―
P17742	Peptidyl-prolyl cis-trans isomerase A	18,0	19	16	17	8	―
Q9Z126	Platelet factor 4	11,2	12	10	21	17	^*^^†^

The amount of PSM (peptide-Spectrum Matches) was used as a quantitative measure of the relative abundance of each protein in the sample [51]. (*) Orthologs detected in human purified platelet releasates [56] or (†) releasates including platelet-derived microparticles [57]. (–) Indicate that the human ortholog has not been detected in platelet secretomes to date. 2C1-: wtC3G; 2C1+: tgC3G; 8A3-: wtC3G∆Cat; 8A3+: tgC3G∆Cat. 15 µl out of 200 µl of platelet secretome obtained from 3 mice of each genotype was used in the proteomic analysis.

### Angiogenesis inhibitors and activators are differentially stored in resting mouse platelets

In human platelets, pro- and anti-angiogenic factors are segregated into distinct subpopulations of α-granules [16, 18]. This prompted us to analyze whether mouse platelets also showed a differential distribution of angiogenic regulators. We examined, by immunofluorescence confocal microscopy, the subcellular localization of two pro-angiogenic factors: VEGF and bFGF, and two anti-angiogenic factors: endostatin and TSP-1 in resting platelets, isolated from tgC3G and tgC3G∆Cat mice and their controls. As in the case of human platelets, most of the mouse platelet α-granules were stained for either VEGF (green) or endostatin (red), as indicated by the lack of colocalization (yellow) in the merged image (Supplementary Figure 2). Similar results were observed when comparing the localization of TSP-1 and bFGF (Supplementary Figure 3).

To our knowledge, this is the first report showing a differential localization of pro- and anti-angiogenic factors in mouse platelet α-granules.

### C3G regulates the release of VEGF, bFGF, endostatin and TSP-1 from mouse platelets following activation with ADP or thrombin

**Figure 1:**
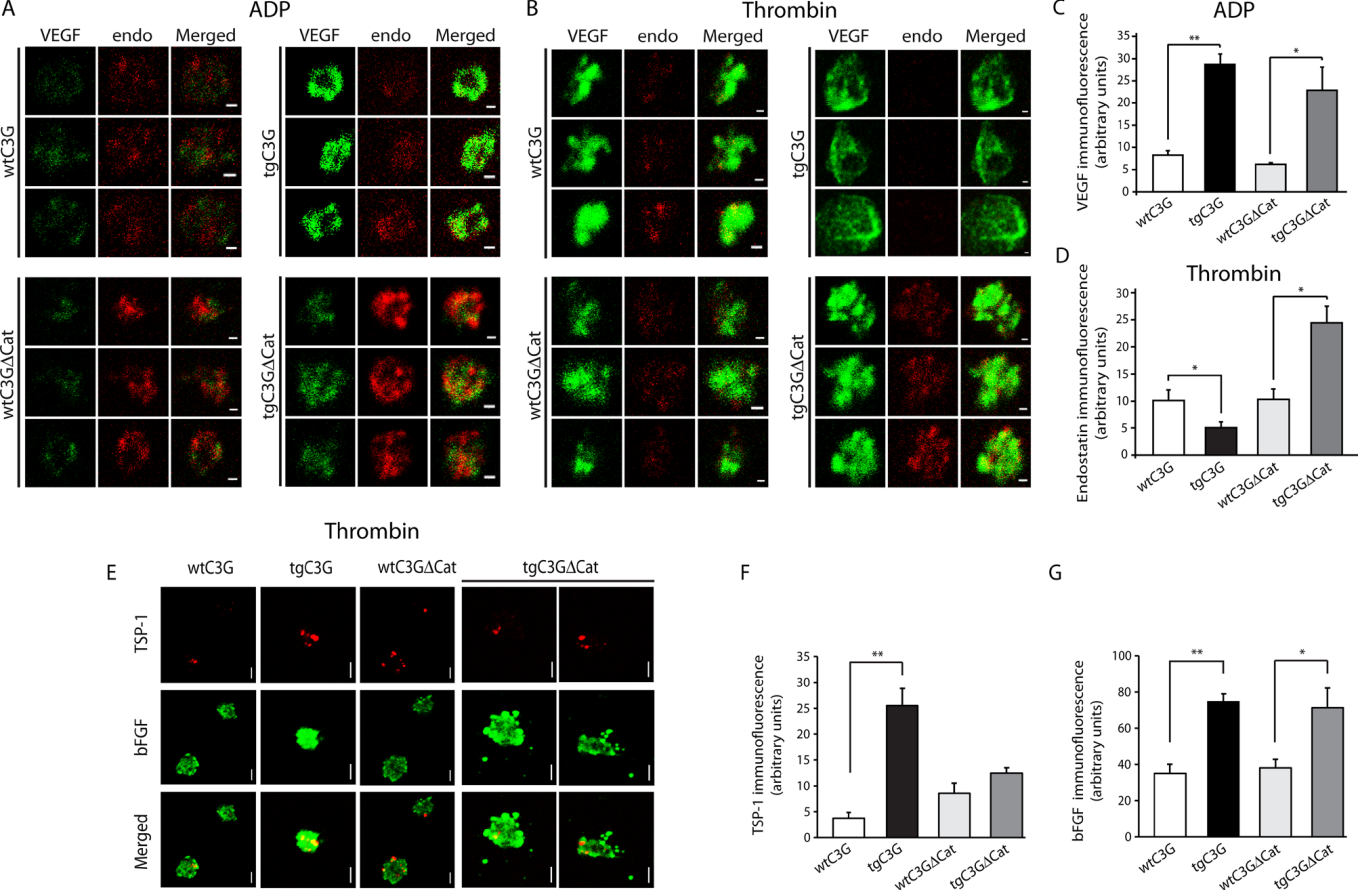
C3G regulates the release of VEGF, bFGF, endostatin and TSP-1 from ADP- and thrombin-stimulated platelets Double immunofluorescence confocal microscopy images showing the subcellular distribution of VEGF (left), endostatin (middle) and an overlay (right) in three representative ADP-activated mouse platelets (**A**) or thrombin-activated mouse platelets (**B**) from each genotype. Platelets were activated for 5 min under stirring conditions. All micrographs were taken at the same exposure time. Scale bars: 0.4 µm. The graphs show arbitrary values of immunofluorescence intensity (mean ± SEM) for VEGF (**C**) or endostatin (**D**) in the ADP- or thrombin-treated platelets respectively. (**E**) Representative confocal microscopy images of the subcellular distribution of TSP-1 and bFGF in thrombin-activated platelets of the indicated genotypes. All micrographs were taken at the same exposure time. Scale bars: 0.4 µm. The graphs show arbitrary values of immunofluorescence intensity (mean ± SEM) for TSP-1 (**F**) or bFGF (**G**) in each genotype. ^*^p < 0.05; ^**^p < 0.01.

Next, we tested whether there is a differential release of pro- and anti-angiogenic factors from mouse platelets activated with ADP or thrombin and whether C3G plays a role. ADP activation promoted the selective release of VEGF- (but not endostatin) containing α-granules from platelets of both control groups (Figure 1A and 1C), in agreement to previous reports from human platelets [18, 27]. However, VEGF was mostly retained within tgC3G and, to a lesser extent, tgC3GΔCat mouse platelets, suggesting a role for C3G as an inhibitor of VEGF secretion (Figure 1A and 1C). Similar results were observed for bFGF, mainly in tgC3G platelets (Supplementary Figure 4). Likewise, we assayed the dynamics of granule release in transgenic mouse platelets activated with thrombin. In human platelets, thrombin activation through PAR4 promotes the differential release of endostatin *versus* VEGF [16]. A similar result was observed in platelets from our control mice (Figure 1B and 1D). However, tgC3G platelets had a lower remaining content of endostatin following activation with thrombin, while tgC3GΔCat platelets seemed to have more remaining endostatin than the controls. In contrast, immunofluorescence analysis of TSP-1 suggests increased retention in tgC3G platelets upon thrombin stimulation (Figure 1E and 1F), in agreement with the proteomic data (Table 1).

Interestingly, thrombin also increased the retention of VEGF and bFGF in both transgenic platelets, mainly in tgC3G (Figure 1E, 1G and Supplementary Figure 4). Images depicted in Supplementary Figures 2 and 3 are the unstimulated controls of those shown in Figures 1A, 1B and Figure 1E, respectively.

All these results suggest that C3G, through GEF- dependent and independent mechanisms, would play a role in mediating a selective release of some angiogenic factors from platelet α-granules in response to thrombin or ADP.

**Figure 2:**
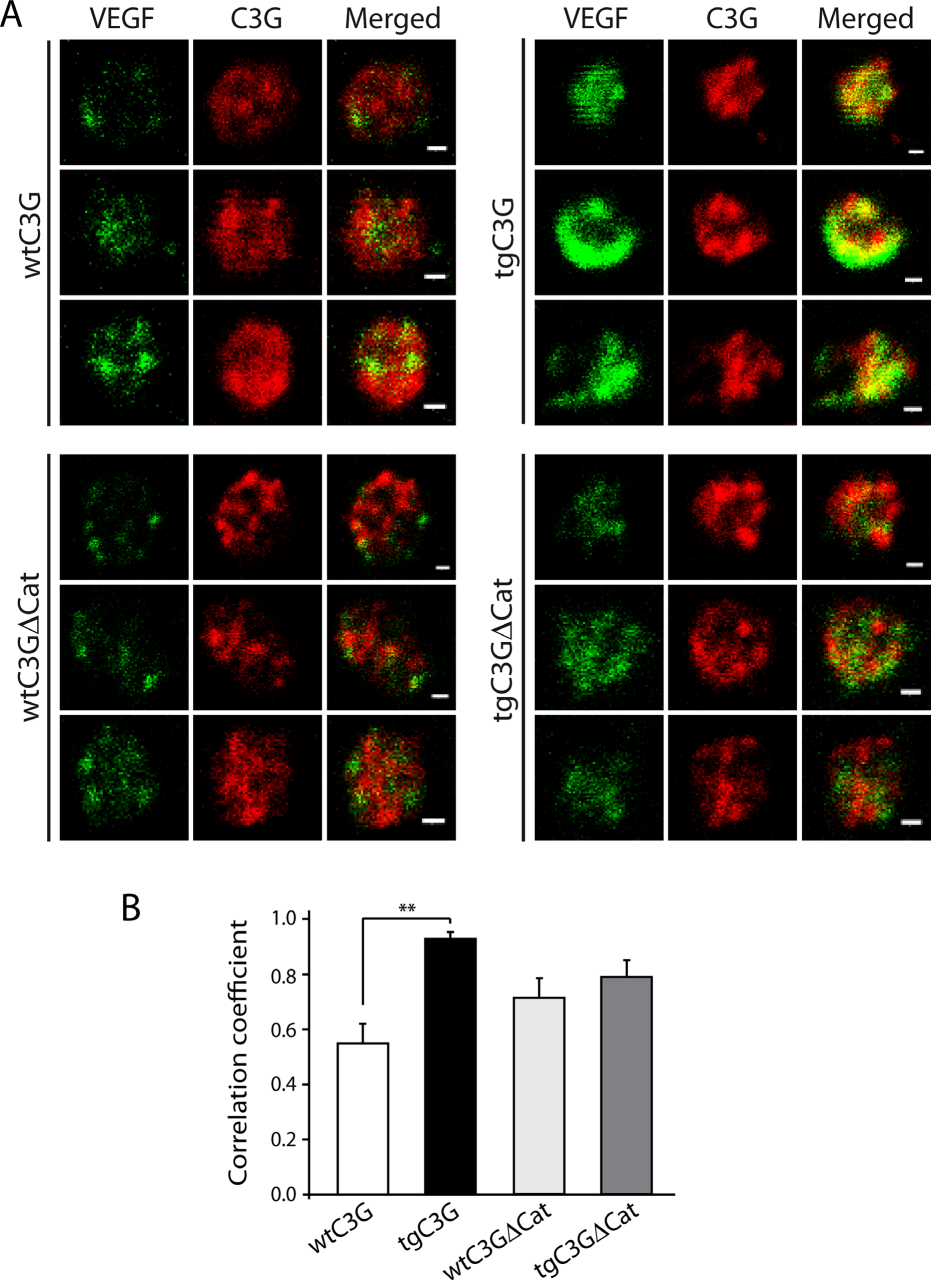
C3G interacts with VEGF in ADP-stimulated platelets from tgC3G mice (**A**) Double immunofluorescence confocal microscopy images showing the subcellular distribution of VEGF (left), C3G (middle) and an overlay (right) in three representative ADP-stimulated mouse platelets from each group. C3G was detected with rabbit anti-C3G antiserum #1008 [33]. All micrographs were taken at the same exposure time. Scale bars: 0.4 µm. (**B**) The graph shows the Manders´correlation coefficients (mean ± SEM) of the colocalization between VEGF and C3G. ^**^p < 0.01.

Results from Figure 1 and Supplementary Figure 4 suggested that in tgC3G platelets and, to a lesser extent, in tgC3G∆Cat platelets, C3G could be mediating the sequestration of VEGF- or bFGF-containing granules. Indeed, following ADP activation, C3G partially colocalized with VEGF, mainly in tgC3G platelets (Figure 2). This result supports a role for C3G as an inhibitor of VEGF release through interaction with VEGF-containing α-granules.

**Figure 3:**
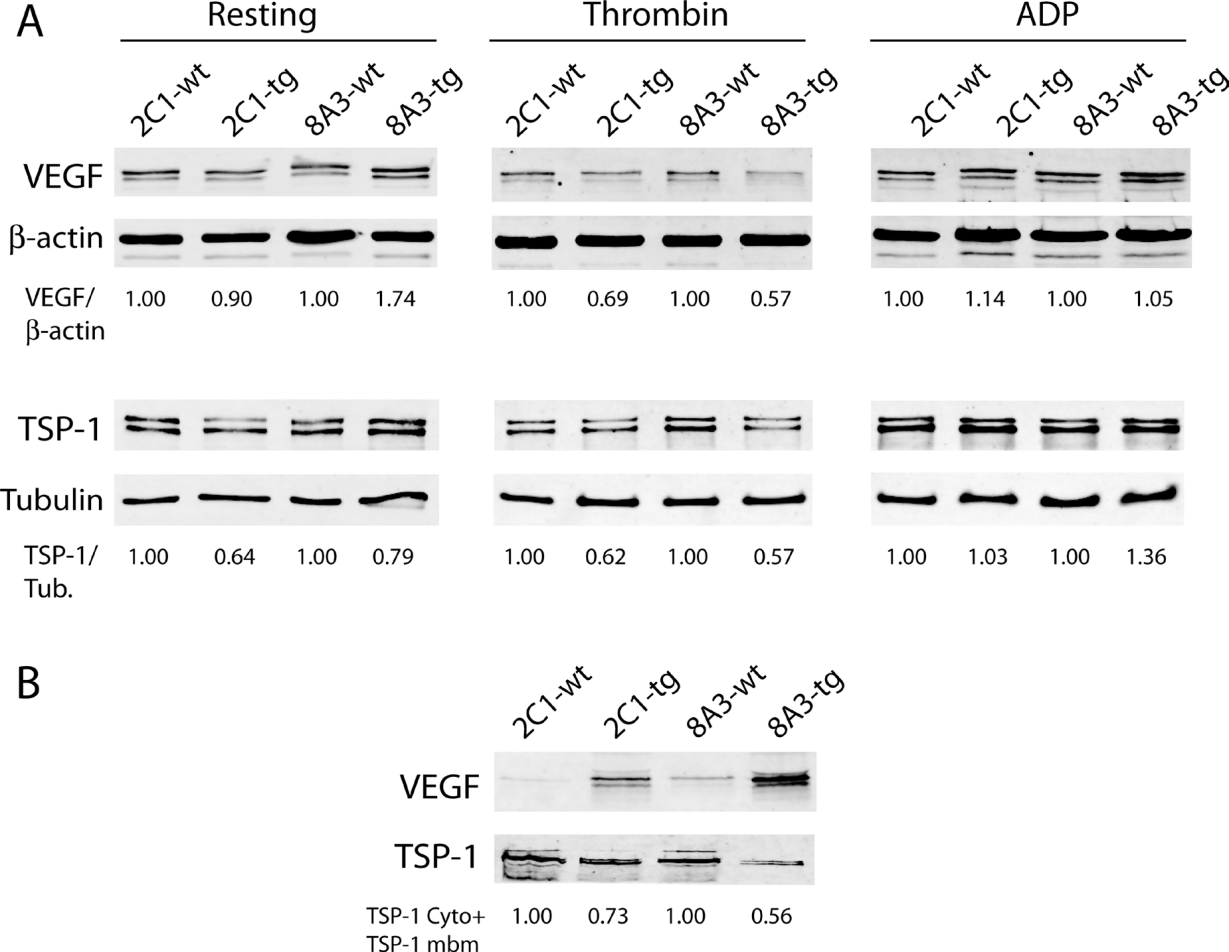
Release of VEGF is impaired in tgC3G and tgC3G∆Cat platelets (**A**) Western blot analysis of VEGF and TSP-1 in cell lysates from mouse platelets of the indicated genotypes (4 mice per group) in resting condition or stimulated with thrombin or ADP. Anti-β-actin (AC-15) and anti-β-tubulin (2-28-33) antibodies from Sigma-Aldrich were used as loading controls. Relative VEGF/β-actin or TSP-1/tubulin ratios are shown. All values are relative to the corresponding wild-type controls. (**B**) Western blot analysis of VEGF and TSP-1 in platelet membranes corresponding to equal amounts of lysed platelets following thrombin activation. 2C1: tgC3G line; 8A3: tgC3G∆Cat line. The total content of TSP-1 (cytoplasm plus membrane) was quantified in thrombin-stimulated platelets of the different genotypes. Values are relative to the corresponding wild-type controls. Cyto: cytoplasm, mbm: membrane.

To further investigate whether C3G regulates VEGF release and the mechanisms involved, we analyzed the amount of protein remaining within the platelet upon stimulation with thrombin or ADP. Unexpectedly, a slight but clear decrease in VEGF was observed in clarified lysates from thrombin-stimulated tgC3G and tgC3G∆Cat platelets, compared to their controls (Figure 3A), suggesting the release of this factor from the transgenic platelets. However, the majority of VEGF was retained on the membrane fraction in both transgenic samples (Figure 3B). These results are in agreement with those from Figures 1 and 2 and support a role for C3G in the regulation of VEGF secretion. Likewise, the content of TSP-1 was lower in lysates from thrombin-stimulated tgC3G and tgC3G∆Cat platelets, compared to their controls (Figure 3A). In addition, this factor was also retained in the plasma membrane of platelets from all genotypes. However, the total amount of TSP-1 (cytosolic content plus membrane content) was lower in the two transgenic samples (Figure 3B), in agreement to the lower secretion detected by proteomics and immunofluorescence. No detectable changes were observed in lysates from ADP-stimulated platelets, probably due to the weakness of this agonist (Figure 3A).

### Releasates from tgC3G and tgC3G∆Cat thrombin- or ADP-activated platelets promote angiogenesis *in vitro*

**Figure 4:**
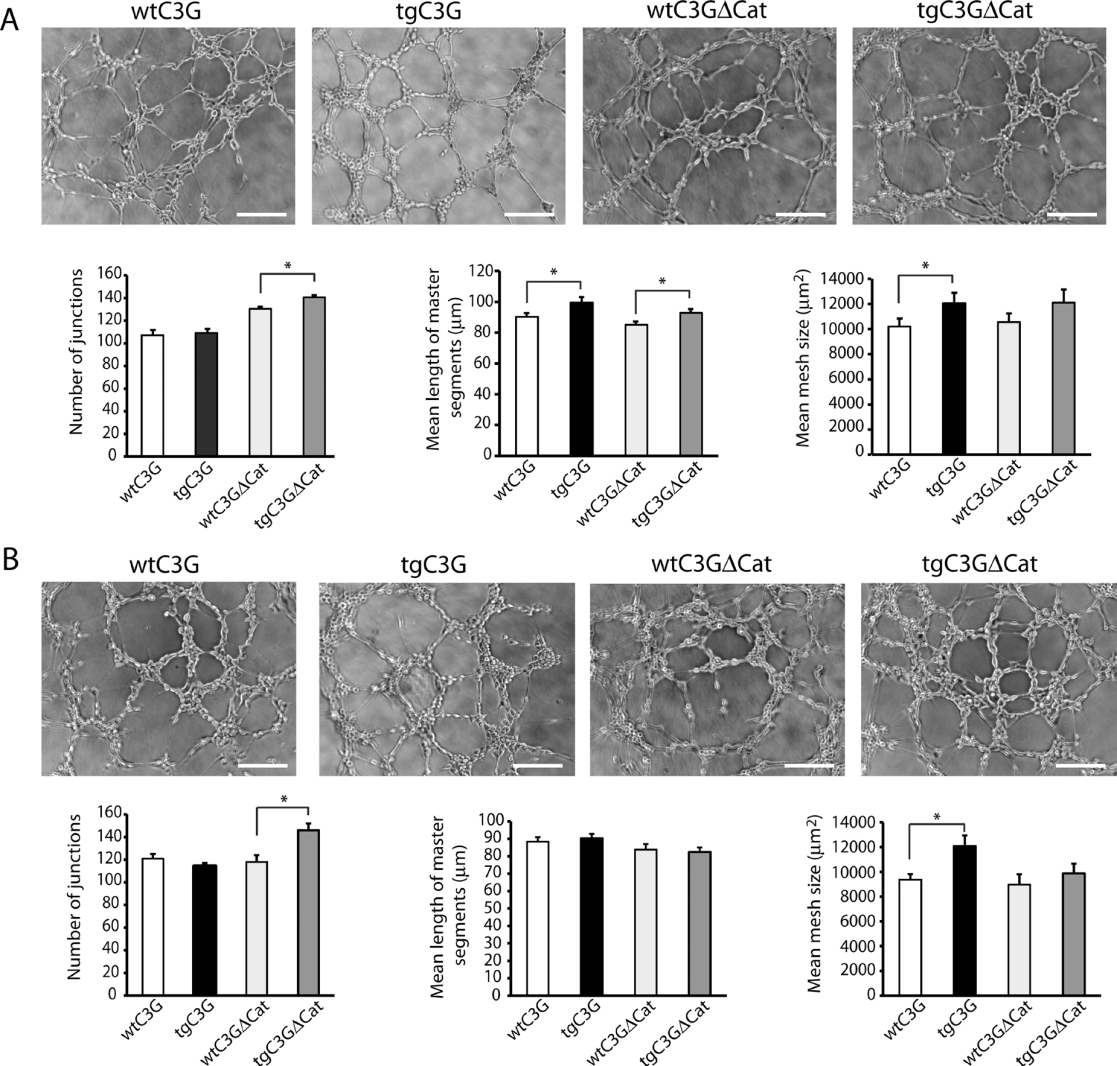
Releasate from thrombin- or ADP-activated transgenic platelets promotes higher formation of capillary networks in HUVECs Representative images showing the capillary-like structures formed by HUVECs seeded onto basement membrane matrix and supplemented with each of the indicated releasates from thrombin- (**A**) or ADP- (**B**) stimulated platelets (2:30 hours post-releasate supplementation). Scale bars: 100 μm. Graphics show the mean values of different network characteristics determined and averaged from 2 to 5 hours. Master segments consist on pieces of tree delimited by two junctions, none exclusively implicated with one branch (master junctions). Data is presented as the mean ± SEM of 3 independent experiments per quadruplicate. ^*^p < 0.05.

To investigate the physiological significance of our findings, we studied the ability of platelet releasates from the different mouse genotypes to promote angiogenesis. To do it, we monitored the formation of capillary tubes in HUVEC exposed to the secretome from platelets stimulated with thrombin or ADP. We quantified 3 parameters: number of junctions, length of master segments, and mesh size to accurately measure capillary formation, as described [28]. Releasates from thrombin-activated transgenic platelets promoted the formation of capillary networks with greater number of junctions, mean length of master segments, and mean mesh size, in comparison with platelets from wild-type mice (being significant, at least, two parameters) (Figure 4A). Similarly, endothelial cells exposed to the secretome from tgC3G and tgC3G∆Cat platelets treated with ADP, showed a significant increase in the formation of capillary networks (Figure 4B). In this case, only the number of junctions in the tgC3G∆Cat and the mean mesh size in the tgC3G reached a significant value, probably due to the weakness of this agonist (less induction of protein release), as compared to thrombin, in agreement with observations by other authors [29]. Overall, these results support the idea of a putative pro-angiogenic role of platelet C3G in response to thrombin and ADP, and are in agreement with the reduced secretion of anti-angiogenic factors detected in both transgenic secretomes in the proteomic analysis. No differences in cell proliferation between HUVECs treated with the different secretomes were observed (data not shown).

### Platelet C3G regulates *in vivo* angiogenesis

**Figure 5:**
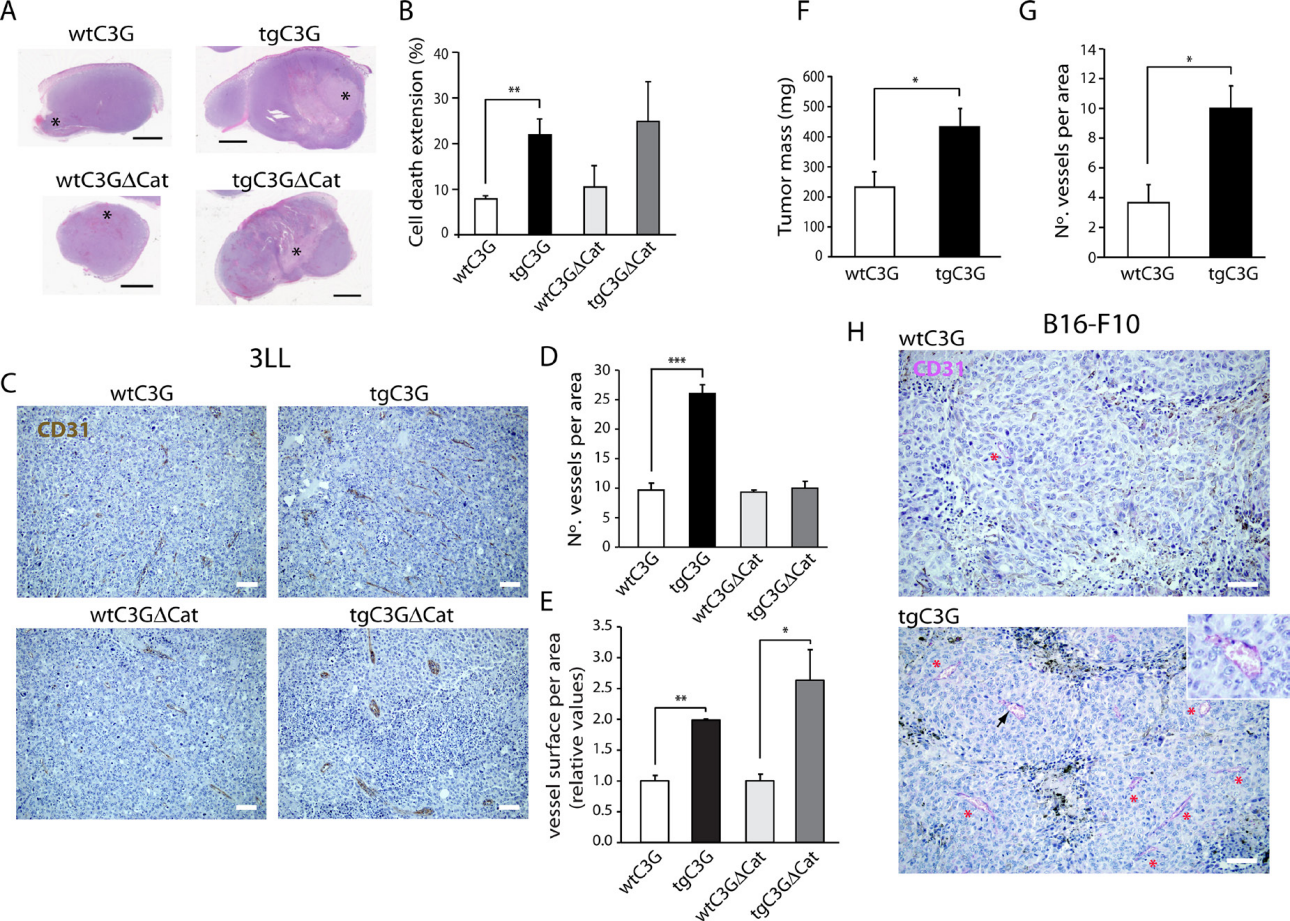
Platelet C3G regulates *in vivo* angiogenesis: Heterotopic tumors display enhanced growth in transgenic mice (**A**) Cell death extension in tumors originated by subcutaneous injection of Lewis lung carcinoma cells in wtC3G, tgC3G, wtC3GΔCat, and tgC3GΔCat mice. Representative light microscopy images of tumor slices stained with hematoxylin/eosin showing cell death area (^*^). (**B**) Quantification of cell death extension is expressed as the percentage of the total tumor area that is occupied by dead cells. The histograms represent the mean value ± SEM (*n =* 4 for wtC3G and tgC3G; *n =* 3 for wtC3GΔCat and tgC3GΔCat). Scale bars: 2.5 mm. (**C**) Representative images of 3LL tumor sections, from the indicated genotypes, showing vessel density by CD31 staining. Scale bars: 20 µm. Quantification of vessel number (**D**) and vessel size (**E**) in tumor sections from the indicated genotypes. 3 representative areas per tumor were analyzed. (F to H) B16-F10 melanoma tumors developed in tgC3G mice have a greater mass and are more vascularized than tumors developed in wtC3G mice. (**F**) Quantification of tumoral mass (*n* = 7 for tgC3G, *n* = 8 for wtC3G). (**G**) Quantification of vessels in 3 representative areas per tumor. All values correspond to the mean ± SEM. (**H**) Representative images of B16-F10 tumor sections showing immunoreactivity for CD31. Presence of vessels is indicated with asterisks. Scale bars: 20 µm. Inset, image enlargement showing a blood vessel, indicated by an arrowhead, stained for CD31 with the Chromo Map kit + Purple. ^*^p < 0.05; ^**^p < 0.01; ^***^p < 0.001.

Platelets cooperate with tumor cells to induce new blood vessels growth. Specifically, tumor cells stimulate the selective release of pro-angiogenic proteins from platelets [18, 30]. Based on that, we analyzed whether platelet C3G regulates the generation of new blood vessels in two models of syngeneic heterotopic tumor cells transplantation: murine Lewis lung carcinoma (3LL) cells and B16-F10 mouse melanoma cells. 3LL-induced tumors presented a higher size accompanied by a higher cell death extension in both transgenic mice (Figure 5A and 5B). These results are indicative of a higher tumorigenic capacity of 3LL cells in mice with circulating tgC3G and tgC3G∆Cat platelets, probably as a consequence of their higher angiogenic potential. Indeed, tumors grown in both transgenic mice showed increased vascularization, as assessed by CD31 staining. TgC3G presented a higher number of vessels, while tgC3G∆Cat had larger vessels than controls (Figure 5C, 5D and 5E). In concordance, we also found a significant higher tumor growth of B16 melanoma cells in tgC3G mice, as compared to their control littermates (Figure 5F). This correlated with a more extensive vascularization (Figure 5G, 5H and Supplementary Figure 5). In addition, tumors developed in tgC3G mice showed a more intense staining for P-selectin, indicating increased platelet activation (Supplementary Figure 5). B16-F10 cells did not grow in tgC3G∆Cat mice, probably due to differences in their genetic backgrounds. Collectively, these results indicate that C3G-regulated platelet secretome favors *in vivo* tumor angiogenesis.

To confirm this *in vivo* pro-angiogenic effect of platelet C3G, angiogenesis was evaluated in a model of oxazolone-induced ear inflammation. No differences in inflammation, measured as ear lobe size, were observed between the different genotypes (data not shown). However, histochemical analysis showed a significantly higher number of blood vessels in the ears from tgC3G mice (Supplementary Figure 6A and 6B), while tgC3G∆Cat mice showed larger vessels than their controls (Supplementary Figure 6A and 6C). These results agree with those observed in the tumor angiogenesis model.

To further characterize the role of platelet C3G in *in vivo* angiogenesis, we evaluated the infiltration of CD31 positive endothelial cells into Matrigel plugs containing bFGF. Only tgC3G∆Cat platelets showed a tendency to induce a greater neovascularization within Matrigel than their controls (data close to statistical significance) (Supplementary Figure 7). These results are not in contradiction with those observed in tumor angiogenesis, taking into account that the mechanisms that operate in both contexts are not comparable. In agreement, no differences in angiogenesis were observed in bFGF-Matrigel plugs implanted in TSP-1 knockout mice *versus* wild types, despite the strong anti-angiogenic potential of TSP-1 [31].

### Platelet C3G promotes melanoma metastasis

The above results indicate that platelet C3G may support tumor cell growth by favoring tumor angiogenesis. Platelets interact with tumor cells to facilitate their metastatic potential *in vivo* [30, 32]. In addition, platelets protects metastatic tumor cells from the immune system during their transit through the circulation and helps tumor cells to attach to the endothelium at metastatic sites [13].

**Figure 6:**
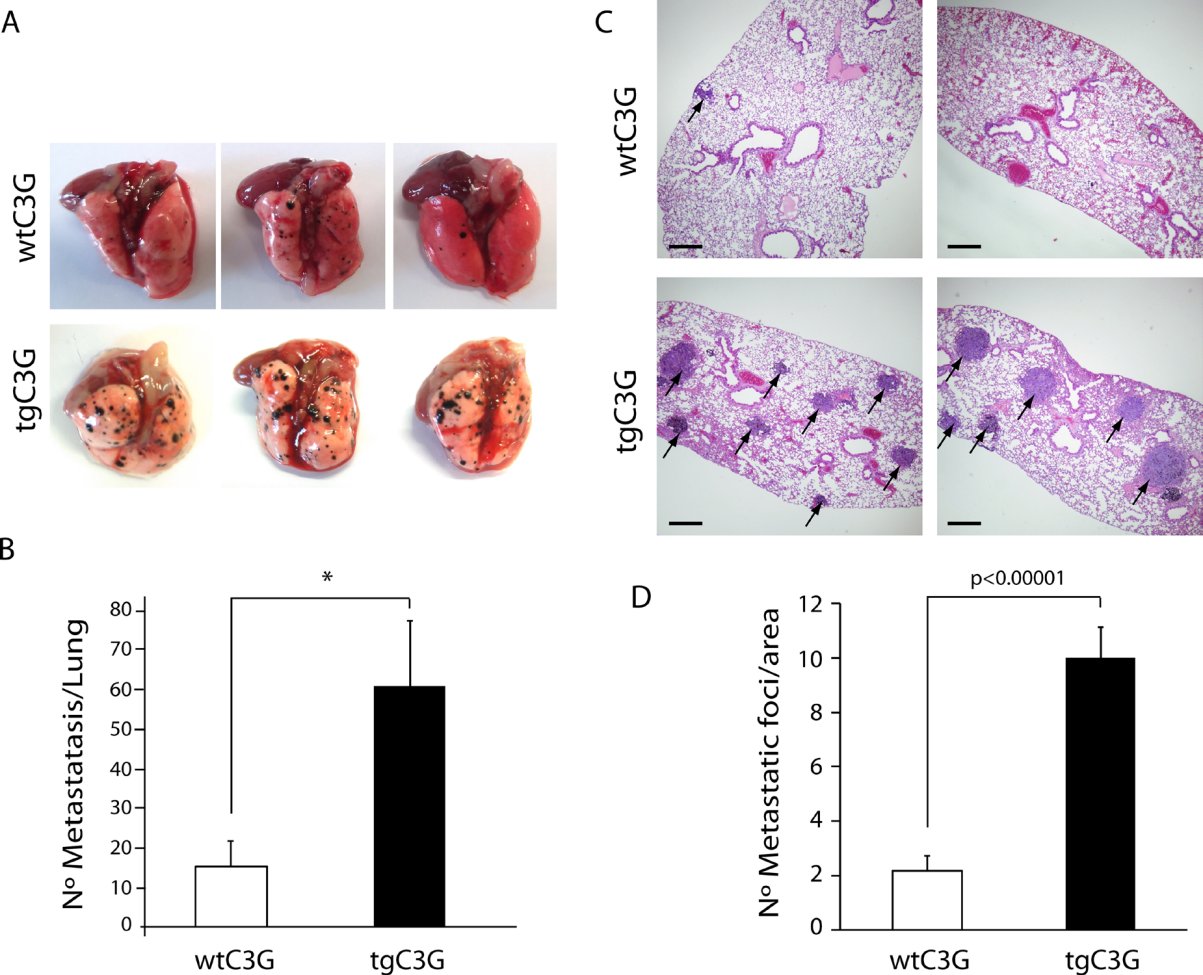
Platelet C3G favors melanoma metastasis TgC3G and wtC3G mice were injected with melanoma B16-F10 cells. Lungs were removed 15 days after tumor cell injection, and surface metastases were quantitated. (**A**) Macroscopic pictures (three representative examples from each of the groups) showing a clear increase in the number of metastatic foci in tgC3G lungs, as compared to lungs from control mice. (**B**) Surface metastases per lung were counted in both groups. The graph shows the average number of metastases in each group (*n* = 4 mice per genotype). ^*^p < 0.05. (**C**) Representative lung sections of two mice from each genotype, showing hematoxylin/eosin staining. Arrows point to metastatic foci in the lung sections. Scale bars: 10 µm. (**D**) Values in the graph represent the number of tumor foci per area (*n* = 12 lung sections per group analyzed). *P*-value comparing the groups is shown.

To investigate whether transgenic expression of C3G in platelets influences the metastatic potential of B16-F10 melanoma cells, we intravenously injected 2.5 x 10^5^ B16-F10 melanoma cells into tgC3G mice and their controls. The average number of metastases in the lungs of tgC3G mice was 61 ± 16.1, whereas the average for the controls was 15.5 ± 7.33 (*n =* 4 mice in both groups, *p =* 0.042) (Figure 6A and 6B). In addition, histological examination of lung sections revealed a significant difference in the number of tumor foci per section in the two experimental groups (Figure 6C and 6D). Lungs from tgC3G mice showed an average of 10 ± 1.12 metastatic foci, while lungs from wild type animals showed 2.17 ± 0.56 foci (*n =* 12 sections analyzed per group, *p =* 0.0000028).

### C3G interacts with Vamp-7 in transgenic mouse platelets

**Figure 7:**
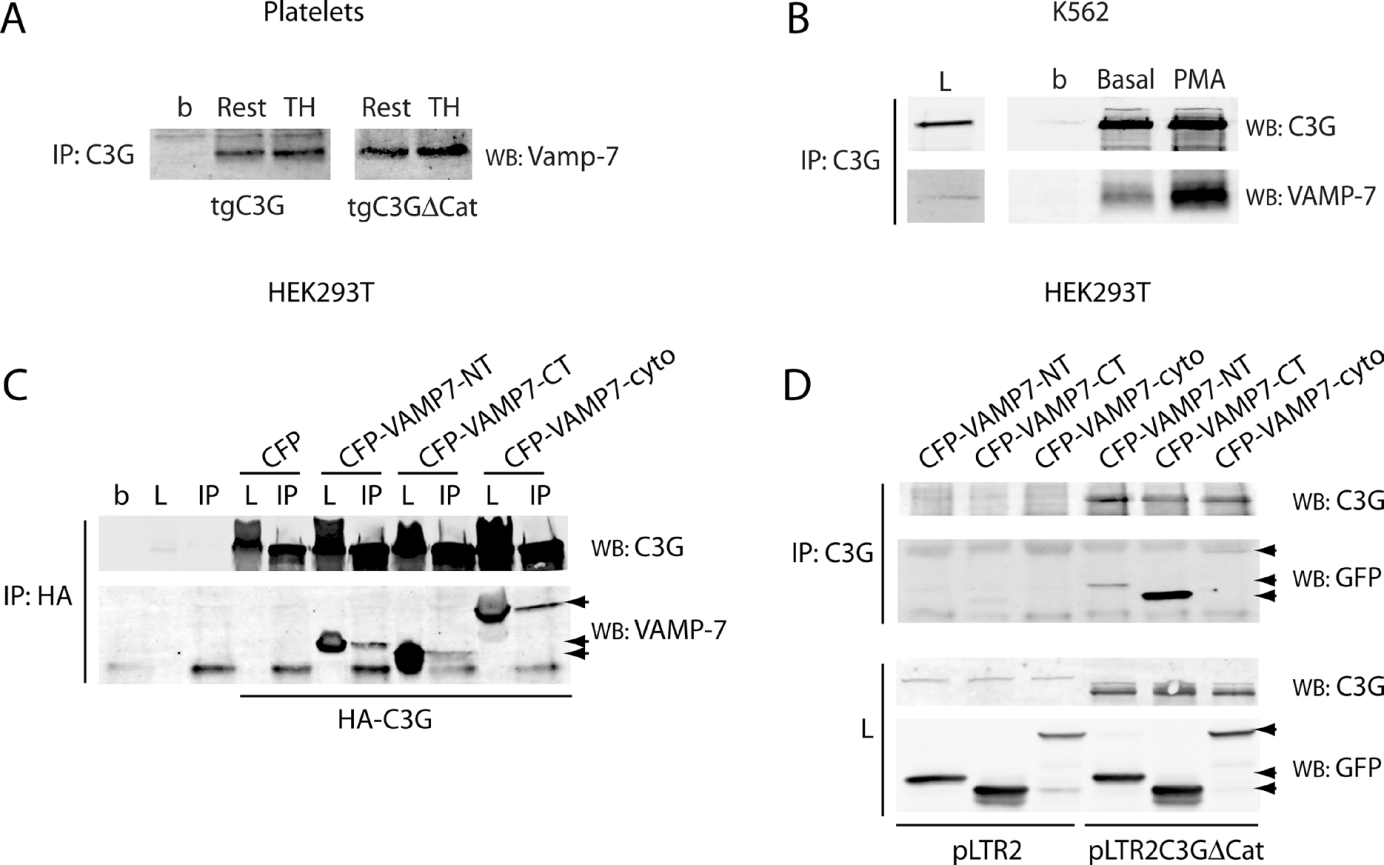
C3G interacts with Vamp-7 in platelets from tgC3G and tgC3G∆Cat mice (**A**) Immunoprecipitation of C3G from resting (Rest) and thrombin (TH, at 0.2 U/mL) stimulated platelets for 5 min at 37°C under stirring. Vamp-7 in the immunocomplexes was detected by Western blot with anti-TI-VAMP antibody (Santa Cruz Biotechnologies, sc-67060). Platelet cell lysate from 4 mice of each genotype was used. b: lysate incubated with agarose beads. (**B**) Immunoprecipitation of C3G from stably C3G-overexpressing K562 cells untreated (basal) or treated with 20 nM PMA for 10 min. C3G and VAMP-7 in the immunocomplexes were detected with anti-TI-VAMP and anti-C3G antibodies. L: total cell lysate (50 µg). b: lysate incubated with agarose beads. (**C**) HEK293T cells were transiently transfected with a HA-tagged C3G construct together with the indicated CFP-VAMP-7 fusion proteins or the empty pCDNA3-CFP-C4 plasmid (CFP). Ectopic C3G was immunoprecipitated with anti-HA.11 monoclonal antibody and C3G and VAMP-7 detected with anti-C3G and anti-TI-VAMP (arrows). b: agarose beads incubated with non-transfected lysate. L: total cell lysate (50 µg). IP: immunoprecipitated with anti-HA.11. (**D**) HEK293T cells were transiently transfected with pLTR2C3G∆Cat construct or the empty pLTR2 vector [33], together with the above CFP-VAMP-7 constructs. C3G was immunoprecipitated with anti-C3G antibodies (IP) and C3G and VAMP-7 detected with anti-C3G and anti-GFP (Santa Cruz Biotechnologies, sc-9996) antibodies. L: total cell lysate. VAMP7-NT: N-terminal (longin) domain of VAMP-7 (aminoacids 1-120); VAMP7-CT: C-terminal (SNARE) domain of VAMP-7 (aminoacids 121-188); VAMP7-cyto: VAMP-7 cytosolic domain (aminoacids 1-188).

It has been described that VEGF is located in VAMP-7 (also known as TI-VAMP)-containing α-granules [23]. VAMP-7 is a v-SNARE protein required for normal a-granule exocytosis [24]. We hypothesize that C3G could retain VEGF by interacting with VAMP-7. To analyze this, we looked for the presence of Vamp-7 in C3G immunoprecipitates from tgC3G and tgC3G∆Cat platelet lysates. Results in Figure 7A support an interaction between Vamp-7 and C3G in both resting and thrombin-activated platelets. This interaction was also detected in a C3G-overexpressing K562 cell line, where the C3G-VAMP-7 association was dependent on PMA, a stimulator of megakaryopoiesis in these cells (Figure 7B). The interaction between C3G and VAMP-7 was further supported by co-immunoprecipitation assays in lysates from HEK293T cells co-transfected with CFP-VAMP-7, together with HA-tagged C3G or pLTR2C3G∆Cat [33] constructs. Both, the longin and the SNARE domains of VAMP-7 seem to contribute to its association with C3G and C3G∆Cat (Figure 7C and 7D). Thus, this interplay between C3G and Vamp-7 might be the mechanism responsible for the retention of these cargos inside the platelet following its activation. The fact that Vamp-7 also interacts with C3G∆Cat provides a potential explanation of how both intact C3G and catalytically inactive C3G affect granule release.

### Platelet spreading is increased in tgC3G and tgC3G∆Cat mouse platelets

**Figure 8:**
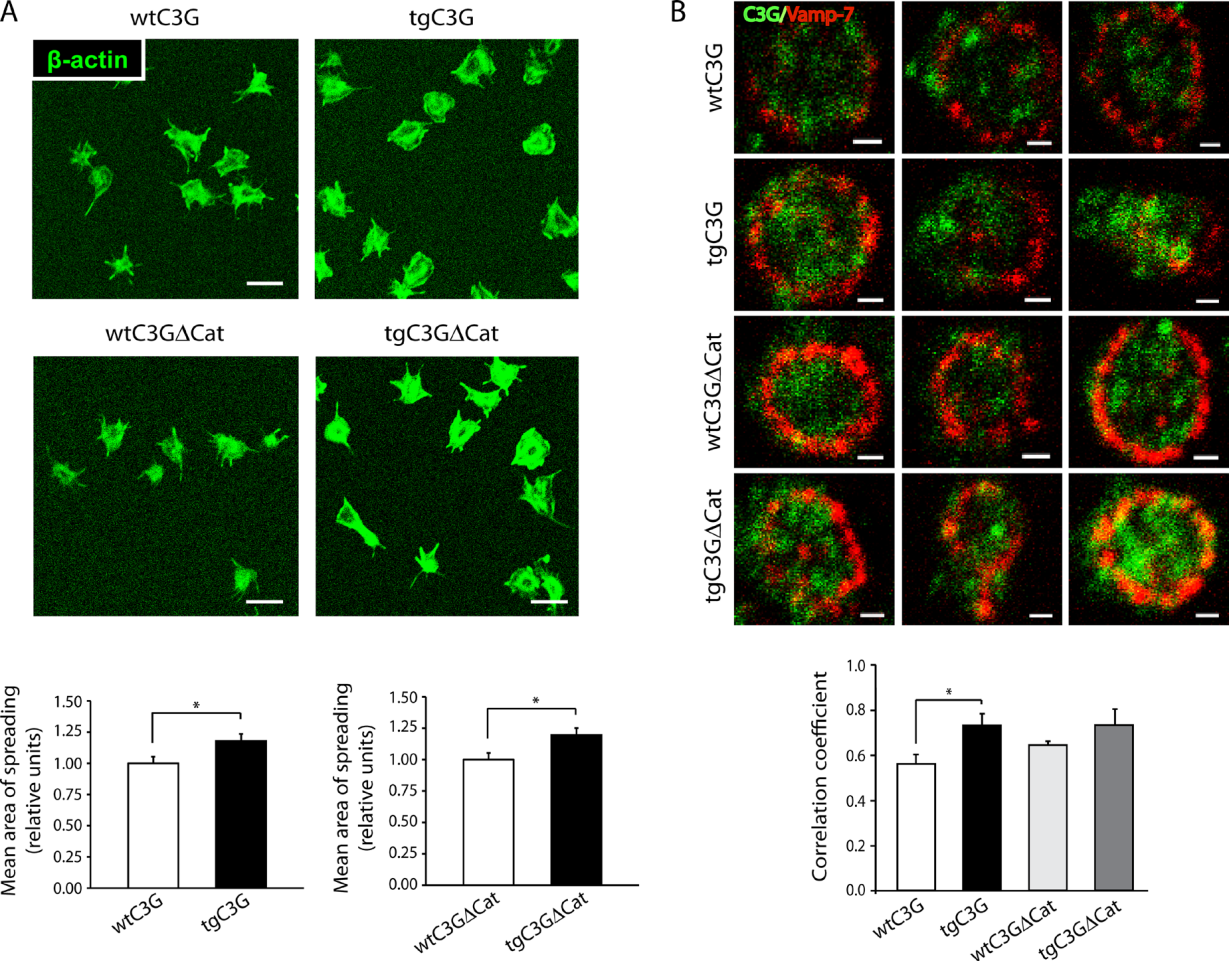
TgC3G and tgC3GΔCat platelets present higher areas of spreading following activation with thrombin, in correlation with higher levels of C3G-Vamp-7 colocalization (**A**) Representative confocal microscopy images of platelet spreading on poly-L-lysine-coated glass coverslips upon stimulation with thrombin. Scale bars: 5 μm. The graphs show the mean ± SEM of the spreading area normalized to that of wild-type platelets. ^*^p < 0.05. An average of 48 platelets from each genotype was measured. (**B**) Representative double immunofluorescence confocal microscopy images of granules in spread platelets stained with antibodies to C3G (rabbit antiserum #1008 [33]) and VAMP-7 (anti-SYBL1, Abcam, ab36195). Scale bars: 2 µm. The graph shows the Manders´correlation coefficients (mean ± SEM) of the colocalization between C3G and Vamp-7. ^*^p < 0.05. TgC3G 6A6 line was used.

Vamp-7 plays a role in the spreading of mouse platelets through its interaction with Varp (VPS9-domain ankyrin repeat protein) and Arp2/3 [24]. Upon activation, Vamp-7-expressing granules are recruited to platelet periphery where they fuse with the plasma membrane, thus contributing to membrane extension during the spreading [23]. We studied the capacity of tgC3G, tgC3G∆Cat and their control platelets to spread on poly-L-lysine upon thrombin stimulation. Spreading, measured as surface area, was significantly increased in both transgenic platelets as compared to that of control platelets (Figure 8A). To link this observation with a functional association between C3G and Vamp-7, we studied their immunocolocalization in spread platelets. Figure 8B shows that C3G colocalized with Vamp-7, mainly in the transgenic platelets, in correlation with the observed increase in spreading (Manders coefficients [M1]: 0.56 ± 0.07 for wtC3G; 0.73 ± 0.09 for tgC3G; 0.65 ± 0.03 for wtC3G∆Cat and 0.73 ± 0.12 for tgC3G∆Cat platelets). Localization of Vamp-7 in the periphery of platelets is an indicator of the proper functioning of the spreading stimulus. No significant colocalization was observed in non-stimulated platelets (data not shown).

These results indicate that C3G plays a role in platelet spreading acting through a GEF independent mechanism and suggest that the interaction between C3G and Vamp-7 increases the capacity of Vamp-7-containing vesicles to fuse with the plasma membrane.

## DISCUSSION

In this study we demonstrate for the first time that transgenic C3G and C3G∆Cat expression modifies platelet α-granule secretion, which leads to changes in the release of angiogenic regulators. As previously observed in human platelets, angiogenic and anti-angiogenic factors are also organized into separate α-granules in mouse platelets [16]. Furthermore, like in human platelets, in wild type mouse platelets ADP induces the release of the angiogenic factors VEGF and bFGF, while thrombin triggers the release of anti-angiogenic factors, such as endostatin and TSP-1 [16, 17]. However, transgenic expression of C3G and mutant C3G∆Cat alters this pattern. Both transgenes caused VEGF and bFGF retention in platelet membranes upon thrombin stimulation, although tgC3G colocalized to a greater extent with both pro-angiogenic proteins. In addition, both transgenes provoked a reduction in the release of several anti-angiogenic factors from ADP- or thrombin-stimulated platelets.

In mouse platelets, not only ADP-, but also thrombin-stimulated platelet secretomes have a net pro-angiogenic effect *in vitro,* promoting capillary formation. This is in agreement with results from human platelets, where thrombin induces the secretion of VEGF and endostatin (through PAR1 and PAR4 respectively), but the global balance is pro-angiogenic in an *in vitro* model [18, 34]. The greater pro-angiogenic effect of the releasates from tgC3G and tgC3G∆Cat platelets, despite VEGF is retained within their α-granules, is in agreement with a non-essential role of VEGF, in *in vitro* angiogenesis [34]. The diminished secretion of anti-angiogenic factors from both transgenic platelets, mainly TSP-1, likely accounts for their increased net pro-angiogenic potential.

This novel function of C3G as a regulator of angiogenesis was confirmed by *in vivo* analyses using two syngeneic heterotopic cancer models, where a significant pro-angiogenic effect of platelet C3G was found. The higher levels of angiogenesis observed in tumors developed in tgC3G and tgC3G∆Cat mice correlates with the lower TSP-1 content in the secretomes from these two genotypes. This is in agreement with the negative role played by platelet TSP-1 in tumorigenesis [12, 35].

A similar tendency was observed in the Matrigel plug assay, but only in tgC3G∆Cat mice. This seemingly discrepancy points to a complex regulatory role for platelet C3G in angiogenesis, beyond granule secretion, which probably involves the participation of cellular interactions between platelets and the surrounding environment.

It is worth mentioning that although tgC3G and tgC3G∆Cat platelets behave similarly in most experimental approaches, they show divergences that may account for the differences observed in the *in vivo* Matrigel assay. Thus, while tgC3G platelets retain more VEGF and bFGF within their α-granules, in response to ADP or thrombin, they also release more endostatin than the other genotypes. Accordingly, elevated levels of endostatin have been linked to various pathological conditions [36]. On the other hand, several reports have documented the important role of the proteolytic activity of the plasminogen (plg) system in Matrigel-induced angiogenesis; e.g. explants of plg-/- mice cultured in Matrigel showed a complete lack of angiogenesis [37, 38]. Plasminogen levels were much lower in the thrombin-induced secretome from tgC3G platelets (Table 1), which may explain the poor angiogenic response of these mice to Matrigel plugs. In contrast, thrombin-induced tgC3G∆Cat secretome presents levels of plasminogen close to control ones. This fact, together with a greater decrease in anti-angiogenic factors, may explain the increased angiogenesis observed in the Matrigel plugs of tgC3G∆Cat mice. In addition, circulating platelets have been described to uptake and store proteins that regulate angiogenesis [12, 25] including VEGF from Matrigel plugs [39]. Subsequently, other mechanisms, such as the uptake of the bFGF from the Matrigel by tgC3G platelets can not be excluded.

Several studies using *in vitro* and *in vivo* models, have demonstrated a correlation between the potential of tumor cells to induce platelet aggregation and the contribution of platelets to tumor metastasis [13, 40, 41]. In this regard, the increase in the number of metastasis observed in tgC3G mice, as compared to their controls, is in agreement with our previous work showing a greater platelet activation and aggregation, both *in vitro* and *in vivo*, in this genotype [9].

Altogether these results indicate that platelet C3G regulates the release of angiogenic factors through GEF-dependent and independent mechanisms, in contrast to its role in the activation of integrin αIIbβ3 that is mediated by Rap1b [9]. Accordingly, it has been described that secretion of VEGF and endostatin may occur independently of the platelet aggregation response [34]. In addition, α-granule secretion, but neither dense granule secretion nor platelet aggregation, is involved in platelet-induced angiogenesis [12].

Our data also unveil a novel interaction between C3G, VEGF and Vamp-7, which could explain the inhibitory role of C3G in the release of VEGF. As VEGF is stored in Vamp-7-containing granules and its secretion is dependent on Vamp-7-mediated exocytosis [23], we reasoned that C3G could prevent VEGF secretion by interacting with Vamp-7. Indeed, C3G colocalizes with VEGF and forms complexes with Vamp-7 in platelets. This interaction was also observed in a K562 cell line over-expressing C3G, which induces megakaryocytic features (unpublished results from our group). Varp, a GEF for Rab21 involved in vesicle transport, regulates the exocytosis of platelet α-granules by interacting with Vamp-7 and ARP2/3 [24]. Similarly to Varp, C3G interacts with both the longin and the SNARE domains of Vamp-7 [42]. Varp inhibits α-granule fusion with the plasma membrane, by preventing SNARE complex formation [24, 43]. Upon activation Varp is released from Vamp-7, which can now interact with t-SNAREs [24]. In contrast, the interaction between C3G and Vamp-7 increases after stimulation, indicating a positive role of C3G in Vamp-7 function. In concordance, transgenic platelets showed higher spreading, suggesting that C3G-Vamp-7 interaction promotes α-granule fusion with the plasma membrane. However, this fusion is not sufficient for the release of VEGF, which rather remains on the plasma membrane. Additional studies should be conducted to determine the mechanism by which VEGF and, presumably bFGF, are being retained by C3G and whether Vamp-7 collaborates. In neuritogenesis, Vamp-7/Arp2/3 complex participates in exocytosis and actin dynamics through an integrin-FAK-Src-dependent pathway [44]. Participation of C3G in focal adhesion complexes and actin cytoskeleton remodeling has been extensively described [3, 6, 7, 45, 46], and supports a role for C3G in Vamp-7-mediated cytoskeletal regulation and membrane delivery.

It should be highlighted that a distinctive feature of C3G, as compared with other GEFs, is its ability to perform a number of functions independently of its Rap1 activating domain. Using our mouse models (tgC3G and tgC3GΔCat) we herein demonstrate that C3G-mediated α-granule release and hence, its role in platelet-mediated angiogenesis are mainly independent of Rap1 functions. In contrast, as previously demonstrated, the role of C3G in platelet aggregation is Rap1-dependent [9]. Moreover, although the results shown here are derived from the analysis of the effect of a transgenic C3G and C3G∆Cat expression, endogenous expression of C3G has been detected in both human and mouse platelets by several authors, using genomic and proteomic approaches [47–49]. This is in agreement with a relevant role of C3G in platelets. In addition, we have preliminary data showing that C3G silencing or knock-out in K562 and HEL erythroid cells prevents the acquisition of megakaryoycytic features (unpublished results).

In summary, we have described a novel function of C3G in platelet-mediated angiogenesis in tumors and other contexts, through the modulation of α-granule secretion, likely by its interaction with Vamp-7. Furthermore, our data suggest that decreased secretion of TSP-1 is probably the most important event that contributes to the tumorigenesis mediated by platelet C3G. Although our proteomic analysis does not show a differential secretion of major pro-tumorigenic growth factors or inflammatory molecules from the transgenic platelets, we can not exclude the possibility that factors, other than angiogenic, may play a role. In addition, we showed a novel role of C3G in platelet-dependent tumor cell metastasis. This points out to a putative prognostic use of platelet C3G in cardiovascular disease and cancer progression. Future studies should aim at addressing whether platelet C3G may play a role in angiogenesis-related disorders and cancer metastasis, which may be of value in designing novel therapeutic approaches.

## MATERIALS AND METHODS

### Animal models

Transgenic mice used in this work have been previously described [9]. C3G (full-length) and C3G∆Cat (deleted in the catalytic region) transgenes from human origin are expressed under the control of the megakaryocyte and platelet specific PF4 promoter. Transgenic C3G (tgC3G) lines 2C1 and 6A6 were used. 2C1 was generally used, unless otherwise indicated. For transgenic C3G∆Cat (tgC3G∆Cat), 8A3 line was used.

### Platelet activation and isolation of platelet releasate

Platelets from 6 mice of the same genotype were pooled and suspended in modified Tyrode´s buffer (130 mM NaCl, 10 mM trisodium citrate, 9 mM NaHCO_3_, 6 mM dextrose, 0.9 mM MgCl_2_, 0.81 mM KH_2_PO_4_, 10 mM Tris pH 7.4) at 2 × 10^8^ platelets/mL, as described [9]. Platelets were supplemented with 2 mM CaCl_2_ and activated under stirring conditions (1100 rpm) with 0.2 U/mL thrombin or 25 mM ADP for 5 min at 37°C. Following aggregation, platelets were removed by centrifuging twice at 2500*g* for 10 min at 4°C and the supernatant harvested.

### LC-MS/MS Analysis and Database Search

Sample proteins were digested using standard methods and analyzed by LC-MS/MS using a LTQ-Velos-Orbitrap mass spectrometer (Thermo Fisher Scientific) coupled to a nano-UPLC system (nanoAcquity, Waters Corp.).

MASCOT search algorithm was used for searching the acquired MS/MS spectra, using Thermo Scientific Proteome Discoverer software (v. 1.4.1.14) against a custom database of mouse proteome AUP000000589 downloaded from UniProt on December 2015, and Mann database of common contaminant sequences. One percent false discovery rate (1% FDR) using Percolator was used for peptide validation [50]. Only proteins with 2 or more peptides were considered. The sum of PSMs (Peptide-Spectrum Matches) was used as a quantitative measure of the abundance of each protein [51].

### Immunofluorescence microscopy

Platelets from 3 mice were pooled and activated as indicated above. Platelets were fixed for 15 minutes by adding 1 vol of 4% formaldehyde, placed onto poly-L-lysine-coated coverslips and allowed to settle for 50 minutes. Attached platelets were permeabilized with 0.5% Triton X100 and blocked overnight in PBS with 1% BSA. Incubation with primary antibodies: VEGF (Abcam, ab1316), bFGF (Abcam ab8880), endostatin (Thermo Scientific, PA1-601), TSP-1 (Thermo Scientific MA5-13398), C3G (#1008 [33]), and VAMP-7 (anti-SYBL1, Abcam, ab36195), was performed at RT for 2h, followed by incubation (1 h at RT) with secondary antibodies: Cy3-conjugated Goat anti-rabbit (Abcam) for bFGF, endostatin and C3G; Cy5-conjugated Goat anti-mouse (Jackson Immunoresearch) for VEGF, TSP-1 and VAMP-7.

Immunofluorescence was quantified with ImageJ software. Colocalization was determined by Manders coefficient (M) analysis, using ImageJ with the JACoP plugin, as described [23, 52].

### Capillary tube formation assay

HUVEC (human umbilical vein endothelial cells) were cultured in EBM medium (Lonza) according to their protocol. Cells were diluted 1:5 in PBS and 32,000 cells (in 37.5 μl volume), mixed with 15 μl of platelet releasate (out of 200 μl, the amount obtained from 3 animals), diluted to 37.5 μl in PBS. The mix (75 μl) was immediately plated onto Matrigel matrix in 96 well plates. Capillary tube formation was allowed to develop at 37°C, 5% CO_2_, and recorded every hour using a Zeiss Axiovert 135 microscope. Angiogenic capacity was determined by quantifying 3 parameters: number of junctions, length of master segments, and mesh size. Data were collected each hour from 2 to 6 hours and represented as the average number of each parameter during the entire experimental time course.

### Heterotopic syngeneic transplantation

A suspension of 10^6^ 3LL (murine Lewis lung carcinoma) cells or 3.3x10^5^ B16-F10 mouse melanoma cells, diluted in 100 µl of PBS, was injected in the shaved flanks of mice. The size of the tumors was measured with a caliper on alternate days and their weight quantified at the time of extraction (day 11 for 3LL tumors, day 15 for B16 tumors). Extracted tumors were fixed in 4% formaldehyde and embedded in paraffin for their histological analysis. The percentage of apoptotic or necrotic cells on tumor sections stained with hematoxylin/eosin was determined using ImageJ software.

### *In vivo* angiogenesis model by oxazolone induction

Mice were pre-sensibilized by subcutaneous injection of a 2% oxazolone (Sigma) solution, prepared in acetone/corn oil (4:1). After six days, contact hypersensitivity was induced by the topical application of a 1% oxazolone solution in one of the mouse ears. The animals were sacrificed 48 h later and treated ears were dissected, fixed in 4% paraformaldehyde and embedded in paraffin. Sections of tissue were stained with hematoxylin/eosin.

### Experimental pulmonary metastasis

B16-F10 mouse melanoma cells (2.5 × 10^5^ in 100 µl PBS per mouse) were injected through the lateral tail vein, as previously described [32]. After 15 days, lungs were removed, rinsed with PBS and the numbers of surface metastases were counted. The pulmonary lobes were fixed in 4% paraformaldehyde, embedded in paraffin and stained using hematoxylin/eosin for their histological analysis.

### Immunohistochemistry

Paraffin embedded sections from 3LL and B16-F10 tumors were used to detect CD31 (Abcam, ab28364), and P-selectin (Santa Cruz Biotechnology, sc-6941) using Ventana Discovery platform (Roche). P-selectin was developed with Chromo Map Kit (Roche) and CD31 with Chromo Map kit (3LL) or Chromo Map kit + Purple (B16-F10). Stained areas were scanned and quantified using ImageJ software.

### Western blot and immunoprecipitation

For Western blot platelets were lysed in RIPA Modified Buffer (100 mM Tris pH 7.5, 400 mM NaCl, 5 mM MgCl_2_, 2% Igepal, 20% Glycerol, 1mM PMSF) supplemented with manufacturer recommended amount of cOmplete™ Protease Inhibitor Cocktail (Roche). After centrifugation, membrane fraction was directly suspended in loading buffer 1X.

For immunoprecipitation, platelets and cell lines were lysed in standard RIPA buffer [3]. Immunocomplexes were pulldown with anti-C3G (Santa Cruz Biotechnologies, sc-15359) or anti-HA.11 (Covance) antibodies and purified with protein G agarose resin 4 rapid run (ABT).

### VAMP-7 and C3G constructs

Human VAMP-7 longin (aa 1-120), SNARE (aa 121-188) and full cytosolic (aa 1-188) domains were amplified from pEGFP-VAMP7 (1-220), a gift from Thierry Galli (Addgene plasmid #42316) [53] and cloned into pcDNA3-CFP-C4 (a gift from Dr. J.M Pereda, IBMCC, Salamanca, Spain) as CFP-fusion proteins, with CFP in the N-terminus. Oligos used were: hVAMP7-F001-EcoRI: 5´-TGAGAATTCATGGCGATTCTTTTTGCTGTTG-3´; hVAMP7-R120-XbaI: 5´-GCGGCCGCTCTAGACTAATTCTCAGAGTGATGCTTCAG-3´; hVAMP7-F121-EcoRI: 5´-TGAGAATTCATGAAGGGCCTAGACAAAGTGATG-3´ and hVAMP7-R188-XbaI: 5´-GCGGCCGCTCTAGACTACTTGAGGTTCTTCATACACATG-3´, based on [43]. An expression vector derived from pCEFL-HA [54], containing HA-tagged full human C3G (with HA in the N-terminus) was a gift from Dr. J.M. Pereda.

### Platelet spreading

Platelets isolated from 4 mice per genotype and suspended in 500 µL of modified Tyrode´s buffer were activated with 0.2 U/mL thrombin. 150 µL of the suspension was immediately applied onto 12-mm glass coverslips precoated with poly-L-lysine. Platelets were allowed to adhere and spread for 30 min at 37°C. Unbound platelets were removed by aspiration, followed by fixation using 4% paraformaldehyde. Platelets were further processed for immunofluorescence as described above. For visualization of actin structures, Oregon Green^®^ 514 Phalloidin (Thermo Fisher Scientific) was used. ImageJ software was used to determine the surface area of spread platelets.

### Matrigel plug angiogenesis assay

The Matrigel plug assay was performed as described [55]. The method is detailed in the Supplemental Methods.

### Statistical analysis

Normality of the data sets was verified using the Kolmogorov-Smirnov test. Homogeneity of the variances was verified by the Levene test. Statistical analysis was performed using an unpaired Student’s *t*-test. Data are represented as mean ± SEM. Results were considered significant when *p <* 0.05.

### Research ethics

This study was carried out in strict accordance with the EU Directive 2010/63/EU for animal experiments http://ec.europa.eu/environment/ chemicals/lab_animals/legislation_en.htm and Uniform Requirements for manuscripts submitted to Biomedical journals http://www.icmje.org. The protocol was approved by the Committee on the Ethics of Animal Experiments of the University of Salamanca, ID number: SAF2010-20918-C02-02. All procedures were performed under isofluorane anesthesia, and all efforts were made to minimize suffering.

## SUPPLEMENTARY MATERIALS FIGURES AND TABLES






