# Supplementary material for: C3G promotes a selective release of angiogenic factors from activated mouse platelets to regulate angiogenesis and tumor metastasis
Source: Oncotarget. 2017 Nov 6;8(67):110994–1011. doi: 10.18632/oncotarget.22339 (PMC5762300; doi:10.18632/oncotarget.22339)
Supplement: Supplementary file 1 [file oncotarget-08-110994-s001.pdf]

C3G promotes a selective release of angiogenic factors from activated mouse platelets to regulate angiogenesis and tumor metastasis

SUPPLEMENTARY MATERIALS

In vivo Matrigel plug angiogenesis assay

Recombinant murine bFGF (Peprotech # 450-33) was added to liquid Matrigel kept on ice at a concentration of 1 mg/mL. Mice were injected subcutaneously near the abdominal midline with ~300 µl of Matrigel, using a 21-gauge needle. After 7 days, mice were euthanized and the Matrigel plugs isolated and fixed in 4% fresh paraformaldehyde solution in PBS for 4 h. Then, the plugs were washed in PBS, cryoprotected in 15% and 30% sucrose solutions, embedded in OCT and frozen in liquid N2-cooled isopentane. Ten µm cryosections were obtained and stored at −20ºC until use. Cryosections were rehydrated in Tris-PBS (TPBS) and blocked for nonspecific binding with 16% sheep sxerum, 1% bovine albumin, and 0.1% Triton X-100 in TPBS (SBT). The primary antibody used was a monoclonal rat anti-mouse CD31 (BD Pharmigen #550274). Single immunofluorescence was performed by incubating the sections with the primary antibody diluted 1/20 in SBT overnight at 4°C, washing in TPBS, and incubating for 1 h with anti-rat Cy5-conjugated secondary antibody (Jackson ImmunoResearch, 712-175-150; 1:100 in TPBS) for 1 hour at room temperature. Nuclei were counterstained with DAPI (Sigma). Negative controls were performed incubating with non-immune rat serum. All images were captured on a Leica SP5 confocal microscope.

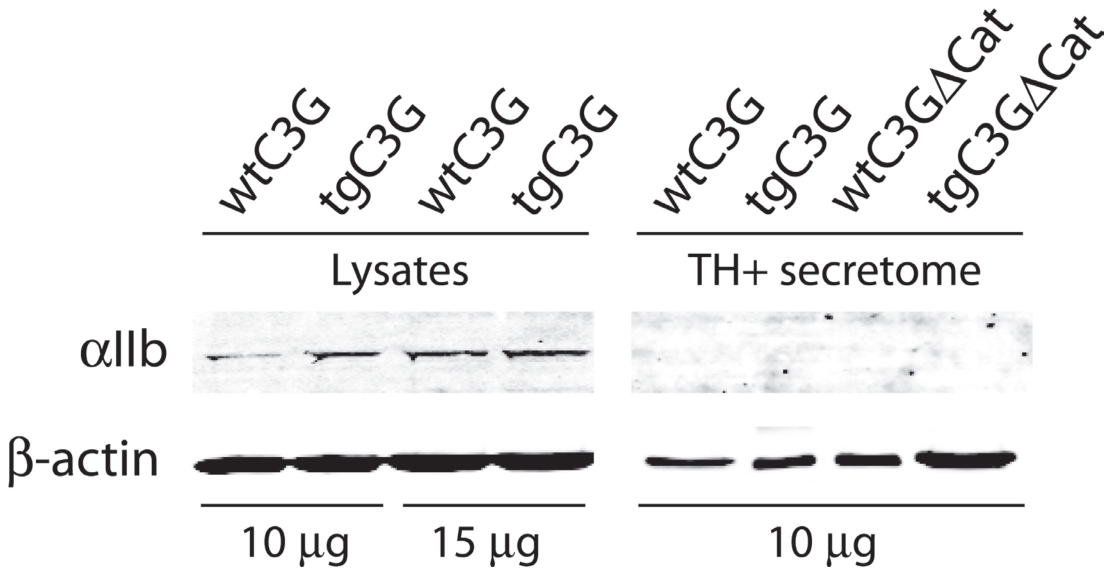

Supplementary Figure 1: Analysis of the purity of platelet secretomes. Western blot analysis of the expression of αIIb integrin subunit in platelet lysates and purified secretomes from thrombin-stimulated platelets (TH+). β-actin expression was used as loading control.

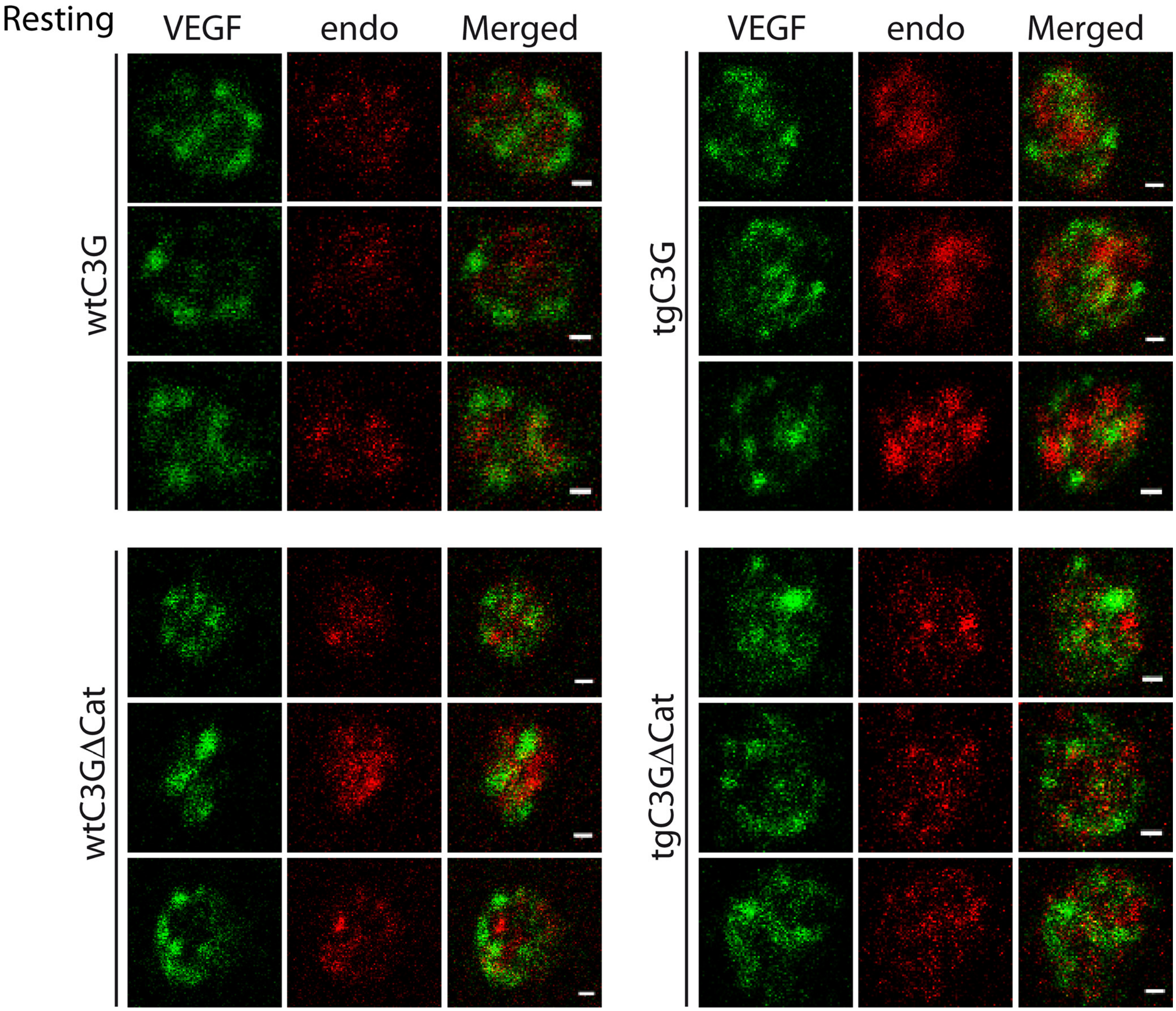

Supplementary Figure 2: VEGF and endostatin are stored in discrete and separate α-granules within resting mouse platelets. Double immunofluorescence confocal microscopy images showing the subcellular distribution of VEGF (left) and endostatin (middle) and an overlay (right) in three representative resting mouse platelets from each of the indicated genotypes. All micrographs were taken at the same exposure time. Scale bars: 0.4 μm.

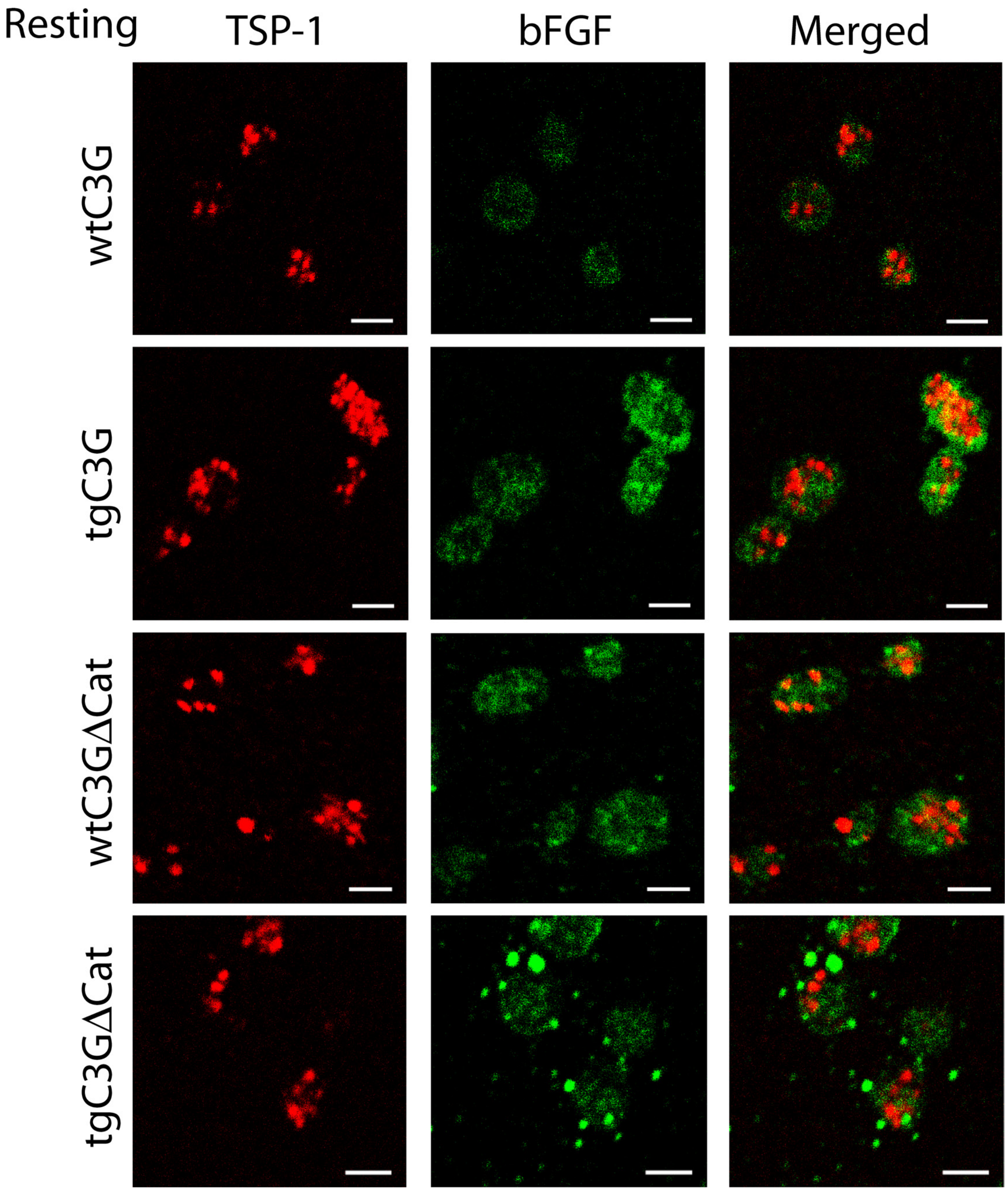

Supplementary Figure 3: bFGF and thrombospondin-1 (TSP-1) are stored in discrete and separate α-granules within resting mouse platelets. Double immunofluorescence confocal microscopy images showing the subcellular distribution of TSP-1 (left) and bFGF (middle) and an overlay (right) in three representative resting mouse platelets from each of the indicated genotypes. All micrographs were taken at the same exposure time. Scale bars: 0.4 µm.

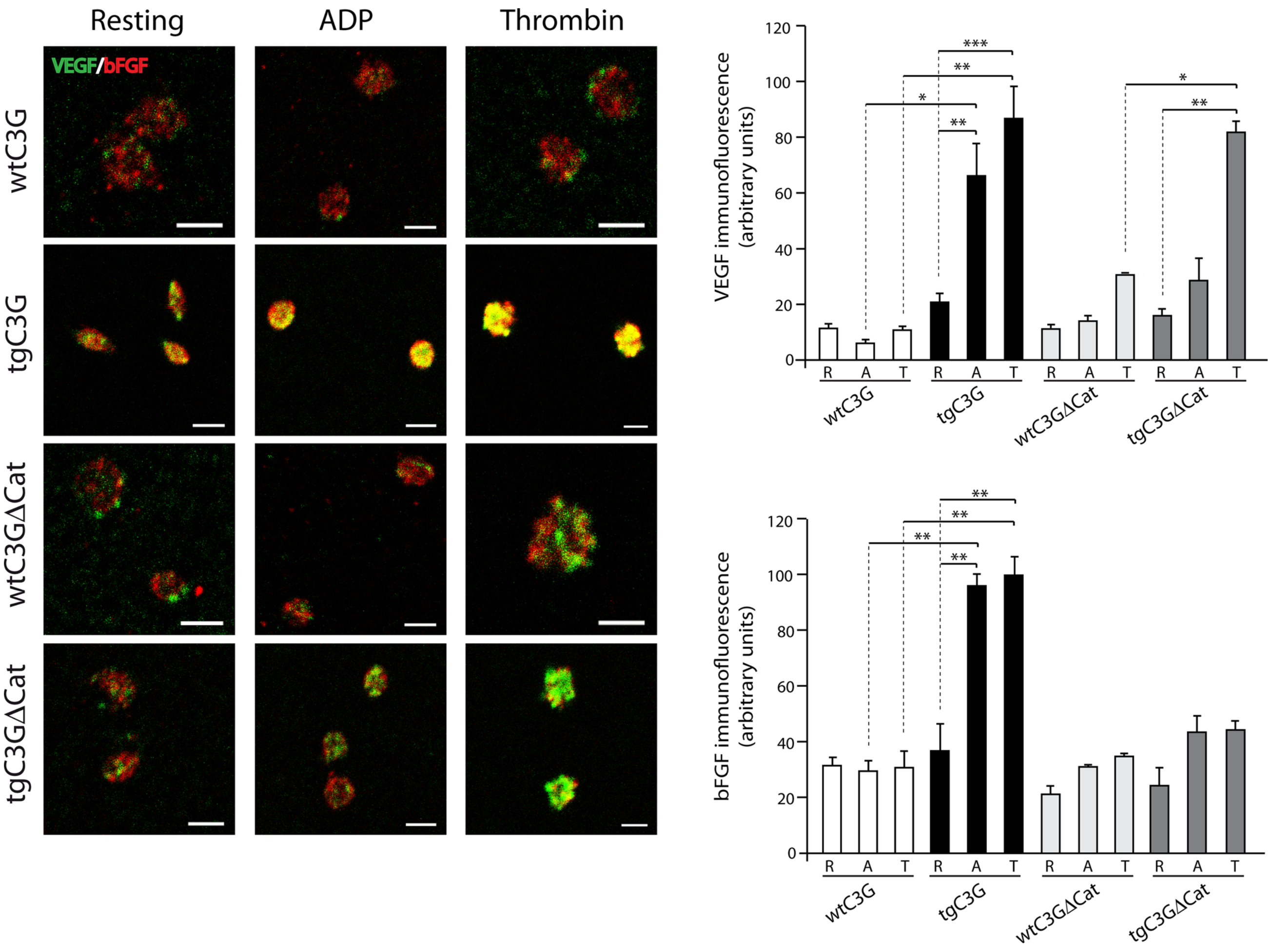

Supplementary Figure 4: VEGF and bFGF are mostly retained within tgC3G and tgC3GΔCat platelets, following activation with ADP or thrombin. Confocal microscopy images are representative of the subcellular distribution of VEGF and bFGF in resting condition, and in ADP- or thrombin-stimulated platelets of the indicated genotypes. All micrographs were taken at the same exposure time. Scale bars: 0.4 µm. The graphs show arbitrary values of immunofluorescence intensity (mean ± SEM) for VEGF (upper graph or bFGF (lower graph) in each genotype and experimental condition: resting (R), stimulated with ADP (A) or thrombin (T). $^{*}p < 0.05$; $^{**}p < 0.01$; $^{***}p < 0.001$.

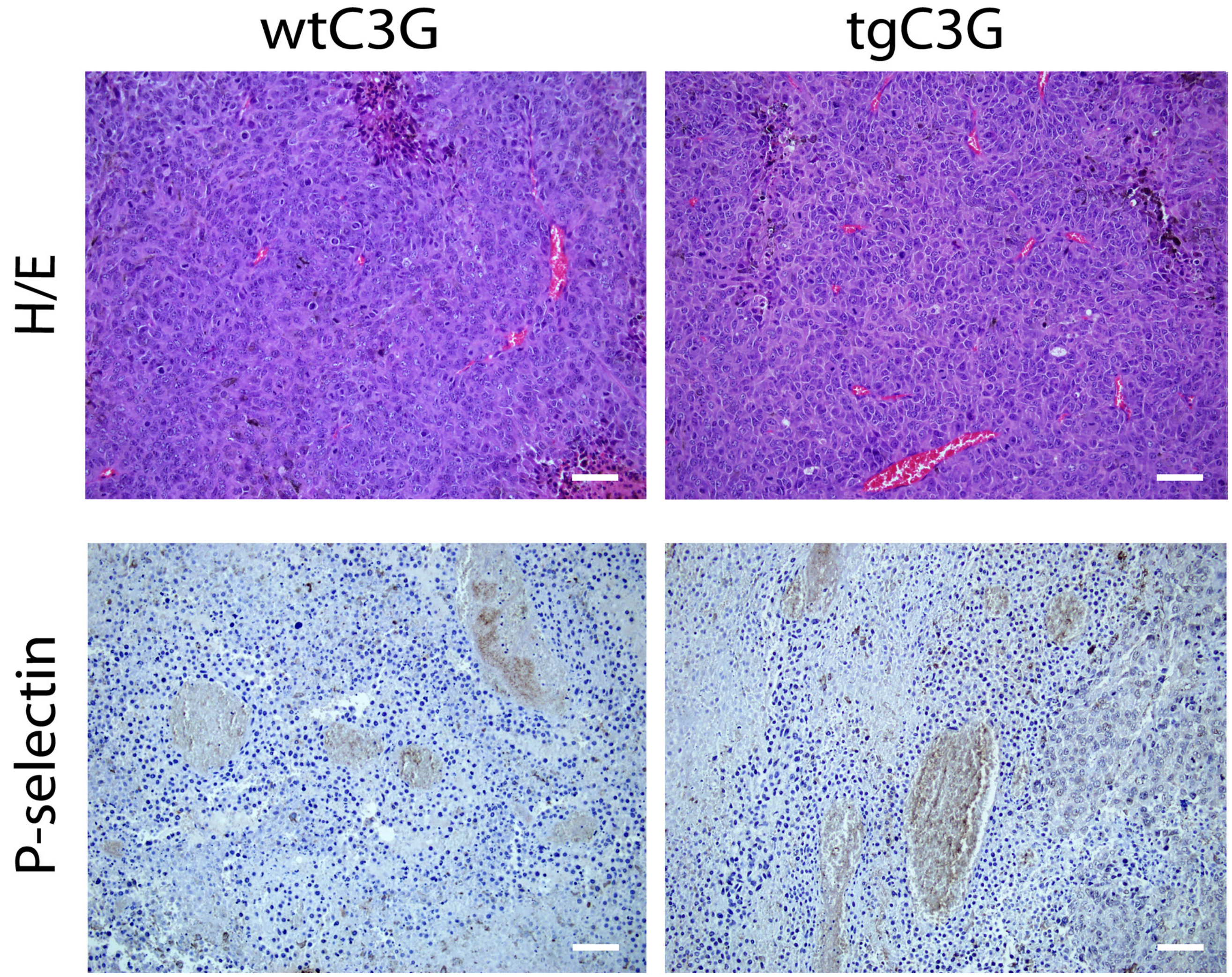

Supplementary Figure 5: B16-F10 cells induced higher angiogenesis in tgC3G mice. Representative images of tumor sections from the indicated genotypes, showing hematoxylin/eosin (H/E) staining and immunoreactivity for P-selectin. Scale bars: 20 µm.

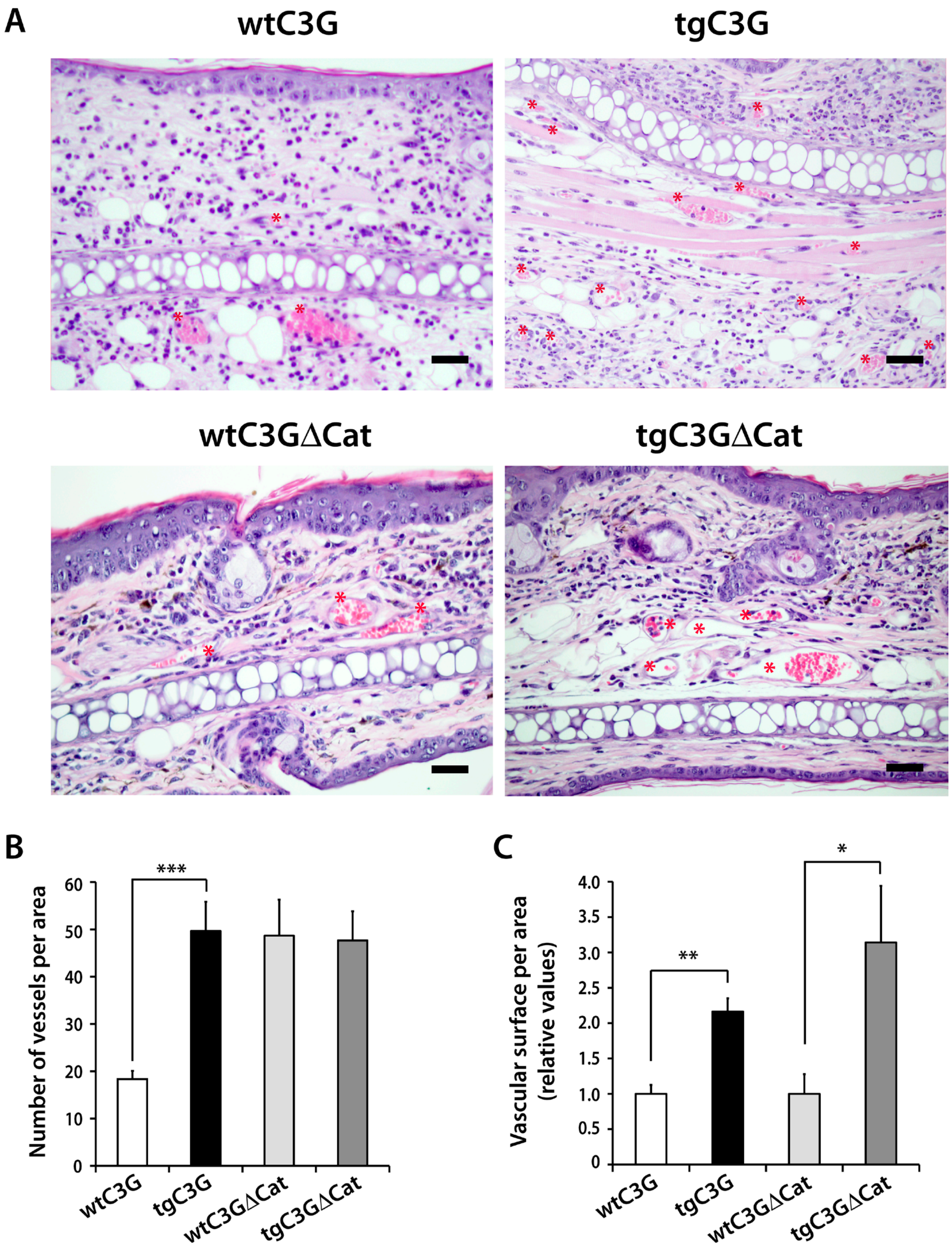

Supplementary Figure 6: Transgenic C3G and C3GΔCat platelets enhance angiogenesis in oxazolone-induced mouse ear inflammation. (**A**) Representative light microscopy images of ear sections stained with hematoxylin/eosin after 48 h of oxazolone treatment. Blood vessels are indicated by red asterisks (*). Scale bars: 20 µm. Quantification of vessel number (**B**) and vessel size (**C**) per area in ear sections from 3 mice per genotype. $^{*}p < 0.05$; $^{**}p < 0.01$; $^{***}p < 0.001$.

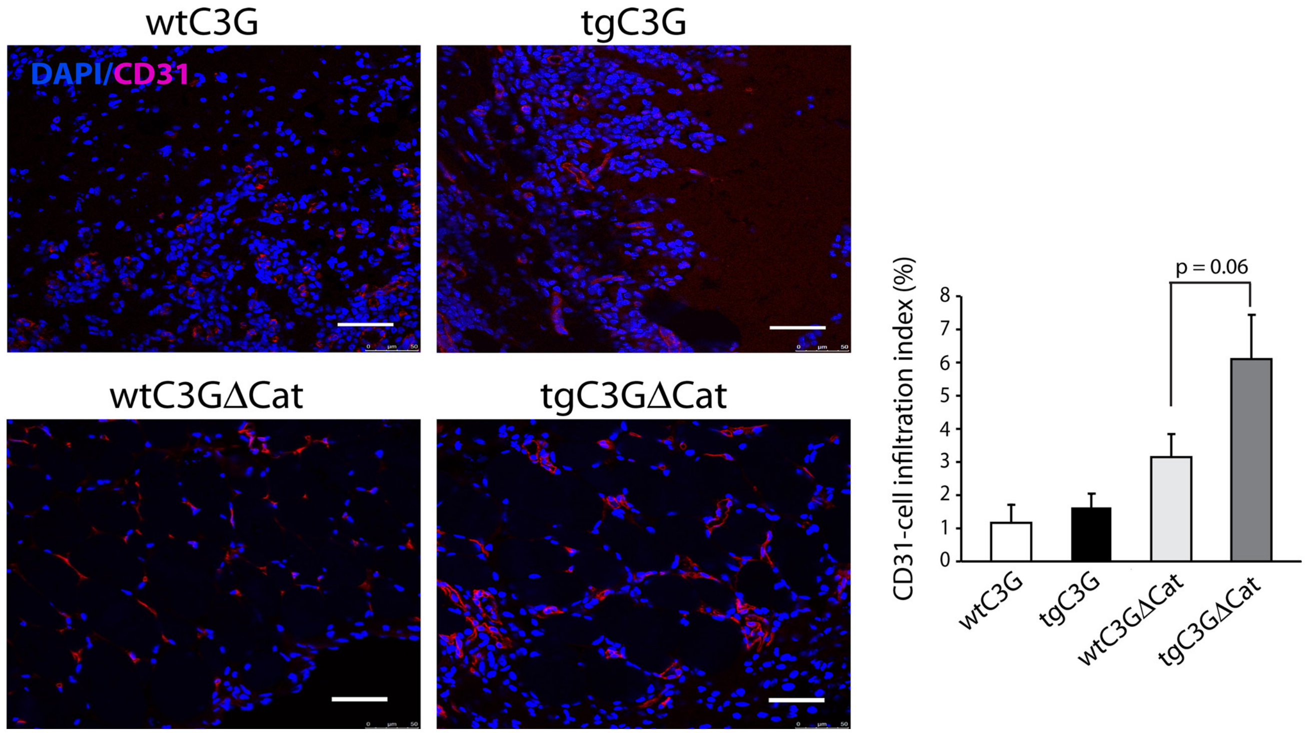

Supplementary Figure 7: Infiltration of CD31-positive endothelial cells into Matrigel plugs containing bFGF. CD31-positive endothelial cells infiltrated into Matrigel plugs subcutaneously injected in wtC3G, tgC3G, wtC3GΔCat and tgC3GΔCat mice. Left: Representative confocal microscopy images showing CD31 positive cells within the Matrigel plugs, 7 days after its injection into mice from each group. Scale bars: 50 µm. Right: Quantification of CD31-positive cell infiltration is expressed as the ratio between the area occupied by the CD31 signal and the area occupied by the DAPI signal. Histograms represent the mean ± SEM (*n* = 4 mice per group, 3–4 measures per mouse).

Supplementary Table 1: Top 100 most abundant proteins released from thrombin-activated mouse platelets. See_Supplementary_Table 1

Supplementary Table 2: Top 100 most abundant proteins released from ADP-activated mouse platelets. See_Supplementary_Table 2
