# Supplementary material for: C3G promotes a selective release of angiogenic factors from activated mouse platelets to regulate angiogenesis and tumor metastasis
Source: Oncotarget. 2017 Nov 6;8(67):110994–1011. doi: 10.18632/oncotarget.22339 (PMC5762300; doi:10.18632/oncotarget.22339)
Supplement: Supplementary file 2 [file oncotarget-08-110994-s002.docx]

**Supplementary Table 1.** **Top 100 most abundant proteins released from thrombin-activated mouse platelets.** The amount of PSM (peptide-Spectrum Matches) was used as a quantitative measure of the relative abundance of each protein in the sample.

**Top 100 2C1-TH**

| **Accession no.** | **Protein identity** | **MW [kDa]** | **WtC3G** | | **TgC3G** | |
| --- | --- | --- | --- | --- | --- | --- |
|  |  |  | **# PSMs** | **Rank** | **# PSMs** | **Rank** |
| P07724 | Serum albumin | 68.6 | 603 | 1 | 635 | 1 |
| Q80YQ1 | Thrombospondin 1 | 129.6 | 194 | 2 | 151 | 2 |
| Q921I1 | Serotransferrin | 76.6 | 129 | 3 | 111 | 3 |
| Q9Z126 | Platelet factor 4 | 11.2 | 99 | 4 | 85 | 4 |
| Q61838 | Alpha-2-macroglobulin | 165.7 | 68 | 5 | 41 | 5 |
| P60710 | Actin. cytoplasmic 1 | 41.7 | 48 | 6 | 37 | 6 |
| P26039 | Talin-1 | 269.6 | 47 | 7 | 21 | 10 |
| P63260 | Actin. cytoplasmic 2 | 41.7 | 47 | 8 | 36 | 7 |
| Q00623 | Apolipoprotein A-I | 30.5 | 47 | 9 | 34 | 8 |
| P01942 | Hemoglobin subunit alpha | 15.0 | 42 | 10 | 26 | 9 |
| E9PV24 | Protein Fga | 87.3 | 39 | 11 | 18 | 13 |
| A8DUK4 | Beta-globin | 15.7 | 37 | 12 | ― | ― |
| A0A075B5P6 | Ig mu chain C region (Fragment) | 50.0 | 30 | 13 | ― | ― |
| Q91X72 | Hemopexin | 51.2 | 28 | 14 | 17 | 14 |
| Q01339 | Beta-2-glycoprotein 1 | 38.5 | 26 | 15 | ― | ― |
| E9QPU1 | von Willebrand factor | 308.9 | 25 | 16 | 12 | 21 |
| P01027 | Complement C3 | 186.3 | 25 | 17 | 6 | 38 |
| A0A0R4J0I1 | MCG1051009 | 46.6 | 24 | 18 | 20 | 11 |
| P20918 | Plasminogen | 90.7 | 22 | 19 | 2 | 92 |
| P21614 | Vitamin D-binding protein | 53.5 | 21 | 20 | 14 | 17 |
| Q8BFZ3 | Beta-actin-like protein 2 | 41.9 | 20 | 21 | ― | ― |
| Q8VDD5 | Myosin-9 | 226.2 | 19 | 22 | 12 | 20 |
| Q9EQI5 | Chemokine (C-X-C motif) ligand 7. isoform CRA_b | 12.2 | 19 | 23 | 20 | 12 |
| Q8K0E8 | Fibrinogen beta chain | 54.7 | 19 | 24 | 5 | 44 |
| P23953 | Carboxylesterase 1C | 61.0 | 16 | 25 | 13 | 19 |
| B7FAV1 | Filamin. alpha (Fragment) | 274.4 | 16 | 26 | 10 | 23 |
| P17182 | Alpha-enolase | 47.1 | 15 | 27 | 9 | 26 |
| P05064 | Fructose-bisphosphate aldolase A | 39.3 | 15 | 28 | 6 | 39 |
| P08905 | Lysozyme C-2 | 16.6 | 14 | 29 | 16 | 15 |
| P28665 | Murinoglobulin-1 | 165.1 | 14 | 30 | 7 | 34 |
| Q00896 | Alpha-1-antitrypsin 1-3 | 45.7 | 13 | 31 | 14 | 18 |
| O08677-2 | Isoform LMW of Kininogen-1 | 47.8 | 13 | 32 | 8 | 30 |
| P17742 | Peptidyl-prolyl cis-trans isomerase A | 17.9 | 12 | 33 | 10 | 22 |
| P68368 | Tubulin alpha-4A chain | 49.8 | 12 | 34 | 9 | 25 |
| P63017 | Heat shock cognate 71 kDa protein | 70.8 | 11 | 35 | 8 | 28 |
| Q64727 | Vinculin | 116.6 | 11 | 36 | 7 | 33 |
| Q8VCM7 | Fibrinogen gamma chain | 49.3 | 11 | 37 | 3 | 71 |
| D3Z5G7 | Protein Ces1b | 62.1 | 11 | 38 | ― | ― |
| P29699 | Alpha-2-HS-glycoprotein | 37.3 | 10 | 39 | 7 | 31 |
| P62962 | Profilin-1 | 14.9 | 10 | 40 | 5 | 49 |
| D6RGQ0 | Complement factor H | 124.9 | 10 | 41 | ― | ― |
| E9Q748 | Antileukoproteinase | 11.9 | 10 | 42 | 3 | 76 |
| O88342 | WD repeat-containing protein 1 | 66.3 | 9 | 43 | 4 | 50 |
| Q60605 | Myosin light polypeptide 6 | 16.9 | 9 | 44 | 3 | 66 |
| G3X9T8 | Ceruloplasmin | 121.0 | 9 | 45 | 6 | 41 |
| A0A075B5V0 | MCG114299 (Fragment) | 12.9 | 8 | 46 | ― | ― |
| P06330 | Ig heavy chain V region AC38 205.12 | 12.9 | 8 | 47 | ― | ― |
| O35930 | Platelet glycoprotein Ib alpha chain | 80.0 | 8 | 48 | 5 | 42 |
| Q8CG19-3 | Isoform 3 of Latent-transforming growth factor beta-binding protein 1 | 147.3 | 8 | 49 | ― | ― |
| P06728 | Apolipoprotein A-IV | 45.0 | 8 | 50 | 7 | 37 |
| Q00897 | Alpha-1-antitrypsin 1-4 | 45.9 | 8 | 51 | 8 | 29 |
| A2AQ07 | Tubulin beta-1 chain | 50.4 | 7 | 52 | 8 | 27 |
| P20029 | 78 kDa glucose-regulated protein | 72.3 | 7 | 53 | 6 | 40 |
| Q9JJZ2 | Tubulin alpha-8 chain | 50.0 | 7 | 54 | 7 | 32 |
| Q00898 | Alpha-1-antitrypsin 1-5 | 45.8 | 7 | 55 | ― | ― |
| P18760 | Cofilin-1 | 18.5 | 7 | 56 | 5 | 46 |
| Q01853 | Transitional endoplasmic reticulum ATPase | 89.2 | 7 | 57 | 4 | 55 |
| D3YY36 | Protein 1300017J02Rik | 68.5 | 7 | 58 | 4 | 52 |
| P21107-2 | Isoform 2 of Tropomyosin alpha-3 chain | 29.0 | 7 | 59 | ― | ― |
| P22599 | Alpha-1-antitrypsin 1-2 | 45.9 | 7 | 60 | 7 | 36 |
| Q07797 | Galectin-3-binding protein | 64.4 | 6 | 61 | 5 | 43 |
| A0A0N4SVL8 | Multimerin-1 | 135.7 | 6 | 62 | ― | ― |
| P20065-2 | Isoform Short of Thymosin beta-4 | 5.0 | 6 | 63 | ― | ― |
| P63101 | 14-3-3 protein zeta/delta | 27.7 | 6 | 64 | 7 | 35 |
| P39876 | Metalloproteinase inhibitor 3 | 24.1 | 6 | 65 | 2 | 84 |
| E9Q7Q3 | Tropomyosin alpha-3 chain | 28.7 | 6 | 66 | ― | ― |
| Q9R0P5 | Destrin | 18.5 | 6 | 67 | ― | ― |
| P09813 | Apolipoprotein A-II | 11.3 | 6 | 68 | 4 | 61 |
| Q02053 | Ubiquitin-like modifier-activating enzyme 1 | 117.7 | 5 | 69 | 4 | 56 |
| P57780 | Alpha-actinin-4 | 104.9 | 5 | 70 | ― | ― |
| Q6IRU2 | Tropomyosin alpha-4 chain | 28.4 | 5 | 71 | 5 | 48 |
| Q64442 | Sorbitol dehydrogenase | 38.2 | 5 | 72 | 5 | 47 |
| P06151 | L-lactate dehydrogenase A chain | 36.4 | 5 | 73 | 4 | 60 |
| Q08761 | Vitamin K-dependent protein S | 74.8 | 5 | 74 | 2 | 78 |
| O88783 | Coagulation factor V | 247.0 | 5 | 75 | 4 | 59 |
| P17751 | Triosephosphate isomerase | 32.1 | 5 | 76 | ― | ― |
| P01029 | Complement C4-B | 192.7 | 5 | 77 | 3 | 67 |
| P68510 | 14-3-3 protein eta | 28.1 | 5 | 78 | ― | ― |
| Q19LI2 | Alpha-1B-glycoprotein | 56.5 | 5 | 79 | ― | ― |
| P52480 | Pyruvate kinase PKM | 57.8 | 4 | 80 | 4 | 51 |
| A0A0R4J1N3 | Apolipoprotein C-III | 10.9 | 4 | 81 | 3 | 62 |
| O89020 | Afamin | 69.3 | 4 | 82 | 4 | 58 |
| F8WI14 | Extracellular matrix protein 1 | 62.6 | 4 | 83 | 2 | 80 |
| Q9D6F9 | Tubulin beta-4A chain | 49.5 | 4 | 84 | 4 | 57 |
| P04202 | Transforming growth factor beta-1 | 44.2 | 4 | 85 | 2 | 88 |
| O70400 | PDZ and LIM domain protein 1 | 35.7 | 4 | 86 | ― | ― |
| Q9QWK4 | CD5 antigen-like | 38.8 | 4 | 87 | ― | ― |
| Q7TQE2 | Zyx protein | 56.9 | 4 | 88 | ― | ― |
| Q03350 | Thrombospondin-2 | 129.7 | 4 | 89 | 2 | 86 |
| A0A0A6YWH7 | Antithrombin-III (Fragment) | 31.9 | 4 | 90 | 2 | 91 |
| A0A075B5P3 | Protein Ighg2b (Fragment) | 36.6 | 4 | 91 | 3 | 63 |
| S4R2J8 | Bridging integrator 2 | 50.8 | 4 | 92 | ― | ― |
| Q91VW3 | SH3 domain-binding glutamic acid-rich-like protein 3 | 10.4 | 4 | 93 | ― | ― |
| Q8K558 | Trem-like transcript 1 protein | 33.5 | 4 | 94 | 2 | 100 |
| H3BJP2 | S-formylglutathione hydrolase (Fragment) | 27.1 | 4 | 95 | 3 | 74 |
| P57774 | Pro-neuropeptide Y | 10.8 | 4 | 96 | 2 | 98 |
| A0A075B5M7 | Protein Igkv5-39 | 10.4 | 4 | 97 | 3 | 68 |
| P27773 | Protein disulfide-isomerase A3 | 56.6 | 4 | 98 | 3 | 65 |
| Q61646 | Haptoglobin | 38.7 | 4 | 99 | ― | ― |
| P50228 | C-X-C motif chemokine 5 | 14.2 | 4 | 100 | ― | ― |
| E9Q223 | Hemoglobin subunit beta-1 (Fragment) | 11.1 | ― | ― | 15 | 16 |
| P01872 | Ig mu chain C region | 49.9 | ― | ― | 9 | 24 |
| I7HJR3 | Beta-2-glycoprotein 1 (Fragment) | 16.8 | ― | ― | 5 | 45 |
| Q99JY9 | Actin-related protein 3 | 47.3 | ― | ― | 4 | 54 |
| Q07456 | Protein AMBP | 39.0 | ― | ― | 4 | 53 |
| O08538 | Angiopoietin-1 | 57.5 | ― | ― | 3 | 72 |
| Q9ESB3 | Histidine-rich glycoprotein | 59.1 | ― | ― | 3 | 75 |
| Q61703 | Inter-alpha-trypsin inhibitor heavy chain H2 | 105.9 | ― | ― | 3 | 70 |
| Q7TMM9 | Tubulin beta-2A chain | 49.9 | ― | ― | 3 | 69 |
| P99024 | Tubulin beta-5 chain | 49.6 | ― | ― | 3 | 73 |
| Q6P1B1 | Xaa-Pro aminopeptidase 1 | 69.5 | ― | ― | 3 | 64 |
| Q9Z1Q5 | Chloride intracellular channel protein 1 | 27.0 | ― | ― | 2 | 93 |
| A0A0A6YWP4 | Complement factor H (Fragment) | 94.2 | ― | ― | 2 | 89 |
| Q8K1B8 | Fermitin family homolog 3 | 75.6 | ― | ― | 2 | 90 |
| P07901 | Heat shock protein HSP 90-alpha | 84.7 | ― | ― | 2 | 85 |
| A0A075B5P4 | Ig gamma-1 chain C region secreted form (Fragment) | 35.7 | ― | ― | 2 | 82 |
| Q9QUM0 | Integrin alpha-Iib | 112.6 | ― | ― | 2 | 87 |
| O55222 | Integrin-linked protein kinase | 51.3 | ― | ― | 2 | 95 |
| P03987-2 | Isoform 2 of Ig gamma-3 chain C region | 36.2 | ― | ― | 2 | 96 |
| Q91XL1 | Leucine-rich HEV glycoprotein | 37.4 | ― | ― | 2 | 81 |
| Q9D154 | Leukocyte elastase inhibitor A | 42.5 | ― | ― | 2 | 83 |
| P09411 | Phosphoglycerate kinase 1 | 44.5 | ― | ― | 2 | 77 |
| Q9DBJ1 | Phosphoglycerate mutase 1 | 28.8 | ― | ― | 2 | 99 |
| P26262 | Plasma kallikrein | 71.3 | ― | ― | 2 | 97 |
| Q99PT1 | Rho GDP-dissociation inhibitor 1 | 23.4 | ― | ― | 2 | 79 |
| D3Z2H9 | Uncharacterized protein | 29.0 | ― | ― | 2 | 94 |

**Top 100 8A3-TH**

| **Accession no.** | **Protein identity** | **MW [kDa]** | **WtC3GΔCat** | | **TgC3GΔCat** | |
| --- | --- | --- | --- | --- | --- | --- |
|  |  |  | **# PSMs** | **Rank** | **# PSMs** | **Rank** |
| P07724 | Serum albumin | 68.6 | 650 | 1 | 790 | 1 |
| Q80YQ1 | Thrombospondin 1 | 129.6 | 163 | 2 | 99 | 3 |
| Q921I1 | Serotransferrin | 76.6 | 127 | 3 | 158 | 2 |
| Q91VB8 | Alpha globin 1 | 15.1 | 73 | 4 | **―** | **―** |
| Q9Z126 | Platelet factor 4 | 11.2 | 71 | 5 | 65 | 7 |
| Q61838 | Alpha-2-macroglobulin | 165.7 | 67 | 6 | 89 | 4 |
| P60710 | Actin, cytoplasmic 1 | 41.7 | 59 | 7 | 29 | 12 |
| P63260 | Actin, cytoplasmic 2 | 41.7 | 57 | 8 | 29 | 11 |
| P26039 | Talin-1 | 269.6 | 55 | 9 | 31 | 10 |
| A8DUK4 | Beta-globin | 15.7 | 54 | 10 | 66 | 6 |
| A0A0R4J0I1 | MCG1051009 | 46.6 | 54 | 11 | 56 | 9 |
| Q00623 | Apolipoprotein A-I | 30.5 | 42 | 12 | 71 | 5 |
| Q8VDD5 | Myosin-9 | 226.2 | 35 | 13 | 12 | 29 |
| E9PV24 | Protein Fga | 87.3 | 31 | 14 | **―** | **―** |
| P20918 | Plasminogen | 90.7 | 30 | 15 | 22 | 17 |
| E9QPU1 | von Willebrand factor | 308.9 | 29 | 16 | 19 | 19 |
| P01027 | Complement C3 | 186.3 | 28 | 17 | 22 | 15 |
| P21614 | Vitamin D-binding protein | 53.5 | 26 | 18 | 26 | 13 |
| P28665 | Murinoglobulin-1 | 165.1 | 26 | 19 | 12 | 30 |
| Q9EQI5 | Chemokine (C-X-C motif) ligand 7, isoform CRA_b | 12.2 | 25 | 20 | 12 | 31 |
| Q91X72 | Hemopexin | 51.2 | 25 | 21 | 23 | 14 |
| B7FAV1 | Filamin, alpha (Fragment) | 274.4 | 22 | 22 | 16 | 22 |
| P23953 | Carboxylesterase 1C | 61.0 | 21 | 23 | 21 | 18 |
| P01872 | Ig mu chain C region | 49.9 | 20 | 24 | 15 | 23 |
| P62962 | Profilin-1 | 14.9 | 19 | 25 | 7 | 50 |
| P29699 | Alpha-2-HS-glycoprotein | 37.3 | 16 | 26 | 18 | 20 |
| Q8CG19 | Latent-transforming growth factor beta-binding protein 1 | 186.5 | 16 | 27 | 6 | 56 |
| P08905 | Lysozyme C-2 | 16.6 | 15 | 28 | 14 | 25 |
| P17742 | Peptidyl-prolyl cis-trans isomerase A | 17.9 | 15 | 29 | 10 | 34 |
| P17182 | Alpha-enolase | 47.1 | 15 | 30 | **―** | **―** |
| P63017 | Heat shock cognate 71 kDa protein | 70.8 | 15 | 31 | 10 | 35 |
| O88342 | WD repeat-containing protein 1 | 66.3 | 14 | 32 | 7 | 46 |
| P68369 | Tubulin alpha-1A chain | 50.1 | 14 | 33 | **―** | **―** |
| Q00896 | Alpha-1-antitrypsin 1-3 | 45.7 | 14 | 34 | 22 | 16 |
| D6RGQ0 | Complement factor H | 124.9 | 14 | 35 | 10 | 38 |
| O08677-2 | Isoform LMW of Kininogen-1 | 47.8 | 14 | 36 | 10 | 36 |
| P68368 | Tubulin alpha-4A chain | 49.8 | 13 | 37 | 9 | 40 |
| Q60605 | Myosin light polypeptide 6 | 16.9 | 13 | 38 | 3 | 99 |
| P09813 | Apolipoprotein A-II | 11.3 | 13 | 39 | 16 | 21 |
| A2AQ07 | Tubulin beta-1 chain | 50.4 | 12 | 40 | 4 | 76 |
| P52480 | Pyruvate kinase PKM | 57.8 | 12 | 41 | 6 | 53 |
| P63101 | 14-3-3 protein zeta/delta | 27.7 | 12 | 42 | 8 | 44 |
| Q00897 | Alpha-1-antitrypsin 1-4 | 45.9 | 12 | 43 | 14 | 26 |
| G3X9T8 | Ceruloplasmin | 121.0 | 12 | 44 | 10 | 37 |
| P22599 | Alpha-1-antitrypsin 1-2 | 45.9 | 12 | 45 | 15 | 24 |
| P20029 | 78 kDa glucose-regulated protein | 72.3 | 11 | 46 | 9 | 39 |
| P05064 | Fructose-bisphosphate aldolase A | 39.3 | 11 | 47 | 13 | 28 |
| O88783 | Coagulation factor V | 247.0 | 11 | 48 | 3 | 88 |
| P52480-2 | Isoform M1 of Pyruvate kinase PKM | 57.9 | 11 | 49 | **―** | **―** |
| Q01339 | Beta-2-glycoprotein 1 | 38.5 | 11 | 50 | 5 | 63 |
| P27773 | Protein disulfide-isomerase A3 | 56.6 | 11 | 51 | 4 | 74 |
| Q9DBD0 | Inhibitor of carbonic anhydrase | 76.7 | 10 | 52 | 14 | 27 |
| Q8VCM7 | Fibrinogen gamma chain | 49.3 | 10 | 53 | 5 | 67 |
| P68372 | Tubulin beta-4B chain | 49.7 | 10 | 54 | **―** | **―** |
| O35930 | Platelet glycoprotein Ib alpha chain | 80.0 | 10 | 55 | 5 | 62 |
| Q8K0E8 | Fibrinogen beta chain | 54.7 | 10 | 56 | **―** | **―** |
| Q00898 | Alpha-1-antitrypsin 1-5 | 45.8 | 9 | 57 | 8 | 45 |
| A0A0N4SVL8 | Multimerin-1 | 135.7 | 9 | 58 | 4 | 72 |
| Q64442 | Sorbitol dehydrogenase | 38.2 | 9 | 59 | 5 | 61 |
| Q01853 | Transitional endoplasmic reticulum ATPase | 89.2 | 8 | 60 | 4 | 81 |
| Q64727 | Vinculin | 116.6 | 8 | 61 | 3 | 95 |
| P18760 | Cofilin-1 | 18.5 | 8 | 62 | 4 | 79 |
| Q07235 | Glia-derived nexin | 44.1 | 8 | 63 | **―** | **―** |
| P17751 | Triosephosphate isomerase | 32.1 | 8 | 64 | 5 | 65 |
| P06728 | Apolipoprotein A-IV | 45.0 | 8 | 65 | 12 | 32 |
| A0A075B5P3 | Protein Ighg2b (Fragment) | 36.6 | 8 | 66 | 6 | 58 |
| Q9WVF5 | Epidermal growth factor receptor | 72.8 | 8 | 67 | 6 | 60 |
| Q02053 | Ubiquitin-like modifier-activating enzyme 1 | 117.7 | 7 | 68 | **―** | **―** |
| P06151 | L-lactate dehydrogenase A chain | 36.4 | 7 | 69 | 4 | 80 |
| Q9WVA4 | Transgelin-2 | 22.3 | 7 | 70 | **―** | **―** |
| P04202 | Transforming growth factor beta-1 | 44.2 | 7 | 71 | **―** | **―** |
| P04186 | Complement factor B | 84.9 | 7 | 72 | 7 | 51 |
| P20065-2 | Isoform Short of Thymosin beta-4 | 5.0 | 7 | 73 | 7 | 48 |
| Q7TQE2 | Zyx protein | 56.9 | 7 | 74 | 3 | 93 |
| P68510 | 14-3-3 protein eta | 28.1 | 7 | 75 | 5 | 71 |
| P57780 | Alpha-actinin-4 | 104.9 | 6 | 76 | **―** | **―** |
| P97315 | Cysteine and glycine-rich protein 1 | 20.5 | 6 | 77 | **―** | **―** |
| O88947 | Coagulation factor X | 53.9 | 6 | 78 | **―** | **―** |
| Q9DBB9 | Carboxypeptidase N subunit 2 | 60.4 | 6 | 79 | 3 | 92 |
| P09411 | Phosphoglycerate kinase 1 | 44.5 | 6 | 80 | **―** | **―** |
| Q6IRU2 | Tropomyosin alpha-4 chain | 28.4 | 6 | 81 | 4 | 75 |
| P99024 | Tubulin beta-5 chain | 49.6 | 6 | 82 | **―** | **―** |
| P21107-2 | Isoform 2 of Tropomyosin alpha-3 chain | 29.0 | 6 | 83 | **―** | **―** |
| Q91VW3 | SH3 domain-binding glutamic acid-rich-like protein 3 | 10.4 | 6 | 84 | 5 | 69 |
| H3BJP2 | S-formylglutathione hydrolase (Fragment) | 27.1 | 6 | 85 | 6 | 59 |
| Q8K558 | Trem-like transcript 1 protein | 33.5 | 6 | 86 | **―** | **―** |
| P08226 | Apolipoprotein E | 35.8 | 6 | 87 | 9 | 41 |
| Q06890 | Clusterin | 51.6 | 6 | 88 | 5 | 66 |
| P29788 | Vitronectin | 54.8 | 6 | 89 | 7 | 52 |
| P08113 | Endoplasmin | 92.4 | 6 | 90 | **―** | **―** |
| A0A0R4J1N3 | Apolipoprotein C-III | 10.9 | 5 | 91 | 7 | 47 |
| Q6YJU1 | Fetuin-B | 33.8 | 5 | 92 | **―** | **―** |
| S4R1B8 | Bridging integrator 2 | 51.5 | 5 | 93 | **―** | **―** |
| Q08761 | Vitamin K-dependent protein S | 74.8 | 5 | 94 | 4 | 77 |
| E9Q7Q3 | Tropomyosin alpha-3 chain | 28.7 | 5 | 95 | 3 | 100 |
| P50396 | Rab GDP dissociation inhibitor alpha | 50.4 | 5 | 96 | **―** | **―** |
| P40142 | Transketolase | 67.5 | 5 | 97 | **―** | **―** |
| P26262 | Plasma kallikrein | 71.3 | 5 | 98 | 7 | 49 |
| P39876 | Metalloproteinase inhibitor 3 | 24.1 | 5 | 99 | **―** | **―** |
| Q9ESB3 | Histidine-rich glycoprotein | 59.1 | 5 | 100 | 3 | 98 |
| P01942 | Hemoglobin subunit alpha | 15.0 | **―** | **―** | 60 | 8 |
| G3X8T9 | Serine (Or cysteine) peptidase inhibitor, clade A, member 3N, isoform CRA_a | 46.7 | **―** | **―** | 11 | 33 |
| Q99K47 | Fibrinogen, alpha polypeptide | 61.2 | **―** | **―** | 8 | 42 |
| P68373 | Tubulin alpha-1C chain | 49.8 | **―** | **―** | 8 | 43 |
| A2A997 | Complement component C8 alpha chain | 65.3 | **―** | **―** | 6 | 54 |
| A0A075B5P4 | Ig gamma-1 chain C region secreted form (Fragment) | 35.7 | **―** | **―** | 6 | 57 |
| Q61703 | Inter-alpha-trypsin inhibitor heavy chain H2 | 105.8 | **―** | **―** | 6 | 55 |
| P01029 | Complement C4-B | 192.7 | **―** | **―** | 5 | 64 |
| Q61129 | Complement factor I | 67.2 | **―** | **―** | 5 | 70 |
| H7BX99 | Prothrombin | 70.1 | **―** | **―** | 5 | 68 |
| O89020 | Afamin | 69.3 | **―** | **―** | 4 | 73 |
| Q19LI2 | Alpha-1B-glycoprotein | 56.5 | **―** | **―** | 4 | 84 |
| B7ZNJ1 | Fibronectin | 239.5 | **―** | **―** | 4 | 78 |
| P03987-2 | Isoform 2 of Ig gamma-3 chain C region | 36.2 | **―** | **―** | 4 | 83 |
| Q91XL1 | Leucine-rich HEV glycoprotein | 37.4 | **―** | **―** | 4 | 82 |
| A0A075B5V1 | Protein Ighv1-31 | 10.9 | **―** | **―** | 4 | 85 |
| P13609 | Serglycin | 16.7 | **―** | **―** | 4 | 86 |
| A0A0A6YX70 | Antithrombin-III (Fragment) | 11.5 | **―** | **―** | 3 | 94 |
| Q07968 | Coagulation factor XIII B chain | 76.1 | **―** | **―** | 3 | 89 |
| P28798 | Granulins | 63.4 | **―** | **―** | 3 | 97 |
| P01660 | Ig kappa chain V-III region PC 3741/TEPC 111 | 12.0 | **―** | **―** | 3 | 90 |
| Q07456 | Protein AMBP | 39.0 | **―** | **―** | 3 | 91 |
| A0A0A6YYE7 | Protein Igkv4-57 (Fragment) | 12.6 | **―** | **―** | 3 | 87 |
| Q6P1B1 | Xaa-Pro aminopeptidase 1 | 69.5 | **―** | **―** | 3 | 96 |
