# Supplementary material for: C3G promotes a selective release of angiogenic factors from activated mouse platelets to regulate angiogenesis and tumor metastasis
Source: Oncotarget. 2017 Nov 6;8(67):110994–1011. doi: 10.18632/oncotarget.22339 (PMC5762300; doi:10.18632/oncotarget.22339)
Supplement: Supplementary file 3 [file oncotarget-08-110994-s003.docx]

**Supplementary Table 2.** **Top 100 most abundant proteins released from ADP-activated mouse platelets.** The amount of PSM (peptide-Spectrum Matches) was used as a quantitative measure of the relative abundance of each protein in the sample.

**Top 100 2C1-ADP**

| **Accession no.** | **Protein identity** | **MW [kDa]** | **WtC3G** | | **TgC3G** | |
| --- | --- | --- | --- | --- | --- | --- |
|  |  |  | **# PSMs** | **Rank** | **# PSMs** | **Rank** |
| P07724 | Serum albumin | 68,6 | 501 | 1 | 684 | 1 |
| Q921I1 | Serotransferrin | 76,7 | 110 | 2 | 117 | 2 |
| Q61838 | Alpha-2-macroglobulin | 165,7 | 81 | 3 | 51 | 4 |
| Q80YQ1 | Thrombospondin 1 | 129,6 | 72 | 4 | 62 | 3 |
| P26039 | Talin-1 | 269,7 | 50 | 5 | 19 | 12 |
| P60710 | Actin, cytoplasmic 1 | 41,7 | 48 | 6 | 24 | 8 |
| P63260 | Actin, cytoplasmic 2 | 41,8 | 46 | 7 | 22 | 10 |
| A0A075B5P6 | Ig mu chain C region (Fragment) | 50,0 | 46 | 8 | ― | ― |
| A0A0R4J0I1 | MCG1051009 | 46,6 | 43 | 9 | 20 | 11 |
| E9PV24 | Protein Fga | 87,4 | 42 | 10 | ― | ― |
| P01027 | Complement C3 | 186,4 | 42 | 11 | 25 | 7 |
| Q91X72 | Hemopexin | 51,3 | 41 | 12 | 24 | 9 |
| Q8K0E8 | Fibrinogen beta chain | 54,7 | 40 | 13 | 7 | 48 |
| Q8VDD5 | Myosin-9 | 226,2 | 38 | 14 | 17 | 14 |
| P21614 | Vitamin D-binding protein | 53,6 | 33 | 15 | 26 | 6 |
| Q8VCM7 | Fibrinogen gamma chain | 49,4 | 30 | 16 | 7 | 45 |
| P20918 | Plasminogen | 90,7 | 30 | 17 | 10 | 32 |
| Q00623 | Apolipoprotein A-I | 30,6 | 29 | 18 | 30 | 5 |
| Q01339 | Beta-2-glycoprotein | 38,6 | 28 | 19 | 12 | 25 |
| A8DUK4 | Beta-globin | 15,7 | 26 | 20 | ― | ― |
| D6RGQ0 | Complement factor H | 125,0 | 26 | 21 | 10 | 31 |
| P63017 | Heat shock cognate 71 kDa protein | 70,8 | 25 | 22 | 12 | 23 |
| G3X9T8 | Ceruloplasmin | 121,0 | 24 | 23 | 9 | 37 |
| P28665 | Murinoglobulin-1 | 165,2 | 24 | 24 | 7 | 47 |
| Q61147 | Ceruloplasmin | 121,1 | 23 | 25 | ― | ― |
| E9QPU1 | von Willebrand factor | 309,0 | 23 | 26 | 9 | 36 |
| B7FAU9 | Filamin, alpha | 280,3 | 21 | 27 | 18 | 13 |
| P05064 | Fructose-bisphosphate aldolase A | 39,3 | 19 | 28 | 11 | 27 |
| P17742 | Peptidyl-prolyl cis-trans isomerase A | 18,0 | 19 | 29 | 16 | 16 |
| P20029 | 78 kDa glucose-regulated protein | 72,4 | 18 | 30 | 12 | 22 |
| O88342 | WD repeat-containing protein | 66,4 | 17 | 31 | 11 | 26 |
| Q9DBD0 | Inhibitor of carbonic anhydrase | 76,7 | 17 | 32 | 9 | 35 |
| P22599 | Alpha-1-antitrypsin 1-2 | 45,9 | 17 | 33 | 12 | 24 |
| Q00897 | Alpha-1-antitrypsin 1-4 | 46,0 | 16 | 34 | 11 | 29 |
| P23953 | Carboxylesterase 1C | 61,0 | 15 | 35 | 13 | 19 |
| P01942 | Hemoglobin subunit alpha | 15,1 | 15 | 36 | 13 | 21 |
| P68368 | Tubulin alpha-4A chain | 49,9 | 15 | 37 | 7 | 50 |
| P68369 | Tubulin alpha-1A chain | 50,1 | 14 | 38 | ― | ― |
| Q00896 | Alpha-1-antitrypsin 1-3 | 45,8 | 14 | 39 | 14 | 18 |
| P63101 | 14-3-3 protein zeta/delta | 27,8 | 14 | 40 | 11 | 28 |
| P06728 | Apolipoprotein A-IV | 45,0 | 14 | 41 | 6 | 61 |
| P29699 | Alpha-2-HS-glycoprotein | 37,3 | 13 | 42 | 6 | 53 |
| Q60605 | Myosin light polypeptide 6 | 16,9 | 13 | 43 | 7 | 49 |
| A2AQ07 | Tubulin beta-1 chain | 50,4 | 13 | 44 | 5 | 64 |
| Q61129 | Complement factor I | 67,2 | 13 | 45 | 3 | 100 |
| P17182 | Alpha-enolase | 47,1 | 13 | 46 | 6 | 56 |
| P21107-2 | Isoform 2 of Tropomyosin alpha-3 chain | 29,0 | 13 | 47 | 8 | 42 |
| Q9QWK4 | CD5 antigen-like | 38,8 | 12 | 48 | 4 | 77 |
| Q9Z126 | Platelet factor 4 | 11,2 | 12 | 49 | 10 | 30 |
| O08677-2 | Isoform LMW of Kininogen-1 | 47,9 | 12 | 50 | 7 | 46 |
| P27773 | Protein disulfide-isomerase A3 | 56,6 | 11 | 51 | 8 | 43 |
| Q61703 | Inter-alpha-trypsin inhibitor heavy chain H2 | 105,9 | 11 | 52 | ― | ― |
| A0A075B5P3 | Protein Ighg2b (Fragment) | 36,7 | 11 | 53 | 5 | 68 |
| O89020 | Afamin | 69,3 | 11 | 54 | 8 | 41 |
| P17751 | Triosephosphate isomerase | 32,2 | 11 | 55 | 8 | 40 |
| P56480 | ATP synthase subunit beta, mitochondrial | 56,3 | 10 | 56 | 4 | 86 |
| G3X8T9 | Serine (Or cysteine) peptidase inhibitor, clade A, member 3N, isoform CRA_a | 46,7 | 10 | 57 | 7 | 51 |
| P52480 | Pyruvate kinase PKM | 57,8 | 10 | 58 | ― | ― |
| P08226 | Apolipoprotein E | 35,8 | 10 | 59 | 5 | 69 |
| Q19LI2 | Alpha-1B-glycoprotein | 56,5 | 10 | 60 | ― | ― |
| P08905 | Lysozyme C-2 | 16,7 | 9 | 61 | 8 | 38 |
| O35930 | Platelet glycoprotein Ib alpha chain | 80,0 | 9 | 62 | 9 | 34 |
| Q02053 | Ubiquitin-like modifier-activating enzyme 1 | 117,7 | 9 | 63 | ― | ― |
| Q00898 | Alpha-1-antitrypsin 1-5 | 45,9 | 9 | 64 | 5 | 70 |
| P68372 | Tubulin beta-4B chain | 49,8 | 9 | 65 | ― | ― |
| P40142 | Transketolase | 67,6 | 9 | 66 | 4 | 74 |
| P62962 | Profilin-1 | 14,9 | 9 | 67 | 4 | 78 |
| Q7TQE2 | Zyx protein | 57,0 | 9 | 68 | 4 | 81 |
| Q9EQI5 | Chemokine (C-X-C motif) ligand 7, isoform CRA_b | 12,2 | 9 | 69 | 10 | 33 |
| P04186 | Complement factor B | 85,0 | 9 | 70 | 4 | 88 |
| Q9ESB3 | Histidine-rich glycoprotein | 59,1 | 9 | 71 | ― | ― |
| P01592 | Immunoglobulin J chain | 18,0 | 9 | 72 | ― | ― |
| A0A075B5V0 | MCG114299 (Fragment) | 12,9 | 8 | 73 | ― | ― |
| Q91XL1 | Leucine-rich HEV glycoprotein | 37,4 | 8 | 74 | 3 | 94 |
| Q9JJZ2 | Tubulin alpha-8 chain | 50,0 | 8 | 75 | ― | ― |
| P68510 | 14-3-3 protein eta | 28,2 | 8 | 76 | 8 | 44 |
| Q61646 | Haptoglobin | 38,7 | 8 | 77 | ― | ― |
| E9Q7Q3 | Tropomyosin alpha-3 chain | 28,7 | 8 | 78 | 8 | 39 |
| A0A075B5V1 | Protein Ighv1-31 | 11,0 | 7 | 79 | ― | ― |
| Q64442 | Sorbitol dehydrogenase | 38,2 | 7 | 80 | 6 | 54 |
| P09411 | Phosphoglycerate kinase 1 | 44,5 | 7 | 81 | ― | ― |
| Q6IRU2 | Tropomyosin alpha-4 | 28,5 | 7 | 82 | 5 | 65 |
| Q9WVF5 | Epidermal growth factor receptor | 72,9 | 7 | 83 | 3 | 97 |
| Q8CG19 | Latent-transforming growth factor beta-binding protein 1 | 186,6 | 7 | 84 | ― | ― |
| Q9DBB9 | Carboxypeptidase N subunit 2 | 60,4 | 7 | 85 | ― | ― |
| P29788 | Vitronectin | 54,8 | 7 | 86 | ― | ― |
| P13020-2 | Isoform 2 of Gelsolin | 80,7 | 7 | 87 | 3 | 98 |
| P01029 | Complement C4-B | 192,8 | 7 | 88 | 4 | 87 |
| P18760 | Cofilin-1 | 18,5 | 7 | 89 | 6 | 55 |
| Q9WVA4 | Transgelin-2 | 22,4 | 7 | 90 | 4 | 79 |
| A2A5N1 | 14-3-3 protein beta/alpha (Fragment) | 18,3 | 7 | 91 | ― | ― |
| P50396 | Rab GDP dissociation inhibitor alpha | 50,5 | 7 | 92 | ― | ― |
| P32261 | Antithrombin-III | 52,0 | 7 | 93 | ― | ― |
| A0A075B5P2 | Protein Igkc (Fragment) | 11,9 | 7 | 94 | ― | ― |
| P01837 | Ig kappa chain C region | 11,8 | 7 | 95 | ― | ― |
| A0A0A6YXA5 | MCG114298 (Fragment) | 12,9 | 6 | 96 | ― | ― |
| S4R1B8 | Bridging integrator 2 | 51,6 | 6 | 97 | ― | ― |
| A0A0R4J1N3 | Apolipoprotein C-III | 10,9 | 6 | 98 | 4 | 73 |
| Q64727 | Vinculin | 116,6 | 6 | 99 | 5 | 67 |
| Q01853 | Transitional endoplasmic reticulum ATPase | 89,3 | 6 | 100 | 4 | 82 |
| P01872 | Ig mu chain C region | 49,9 | ― | ― | 16 | 15 |
| E9Q223 | Hemoglobin subunit beta-1 (Fragment) | 11,1 | ― | ― | 15 | 17 |
| Q99K47 | Fibrinogen, alpha polypeptide | 61,3 | ― | ― | 13 | 20 |
| P68373 | Tubulin alpha-1C chain | 49,9 | ― | ― | 7 | 52 |
| A0A075B5M7 | Protein Igkv5-39 | 10,4 | ― | ― | 6 | 57 |
| A0A075B5P4 | Ig gamma-1 chain C region secreted form (Fragment) | 35,7 | ― | ― | 6 | 58 |
| P20065-2 | Isoform Short of Thymosin beta-4 | 5,0 | ― | ― | 6 | 59 |
| P06151 | L-lactate dehydrogenase A chain | 36,5 | ― | ― | 6 | 60 |
| Q91VW3 | SH3 domain-binding glutamic acid-rich-like protein 3 | 10,5 | ― | ― | 6 | 62 |
| P68254-2 | Isoform 2 of 14-3-3 protein theta | 27,7 | ― | ― | 6 | 63 |
| A0A0R4J1P2 | Tropomyosin alpha-3 chain | 32,8 | ― | ― | 5 | 66 |
| Q9CQV8 | 14-3-3 protein beta/alpha | 28,1 | ― | ― | 5 | 71 |
| P09813 | Apolipoprotein A-II | 11,3 | ― | ― | 5 | 72 |
| P06330 | Ig heavy chain V region AC38 205.12 | 12,9 | ― | ― | 4 | 75 |
| Q9R0P5 | Destrin | 18,5 | ― | ― | 4 | 76 |
| H3BJP2 | S-formylglutathione hydrolase (Fragment) | 27,1 | ― | ― | 4 | 80 |
| P61982 | 14-3-3 protein gamma | 28,3 | ― | ― | 4 | 83 |
| P03987-2 | Isoform 2 of Ig gamma-3 chain C region | 36,2 | ― | ― | 4 | 84 |
| A0A0A6YX70 | Antithrombin-III (Fragment) | 11,6 | ― | ― | 4 | 85 |
| P08249 | Malate dehydrogenase, mitochondrial | 35,6 | ― | ― | 4 | 89 |
| P01660 | Ig kappa chain V-III region PC 3741/TEPC 111 | 12,1 | ― | ― | 3 | 90 |
| Q8CG19-3 | Isoform 3 of Latent-transforming growth factor beta-binding protein 1 | 147,3 | ― | ― | 3 | 91 |
| F8WI14 | Extracellular matrix protein 1 | 62,7 | ― | ― | 3 | 92 |
| Q07456 | Protein AMBP | 39,0 | ― | ― | 3 | 93 |
| P97315 | Cysteine and glycine-rich protein 1 | 20,6 | ― | ― | 3 | 95 |
| Q9Z2U1 | Proteasome subunit alpha type-5 | 26,4 | ― | ― | 3 | 96 |
| P97447 | Four and a half LIM domains protein 1 | 31,9 | ― | ― | 3 | 99 |

**Top 100 8A3-ADP**

| **Accession no.** | **Protein identity** | **MW [kDa]** | **WtC3GΔCat** | | **TgC3GΔCat** | |
| --- | --- | --- | --- | --- | --- | --- |
|  |  |  | **# PSMs** | **Rank** | **# PSMs** | **Rank** |
| P07724 | Serum albumin | 68,6 | 616 | 1 | 685 | 1 |
| Q921I1 | Serotransferrin | 76,7 | 115 | 2 | 120 | 2 |
| Q80YQ1 | Thrombospondin 1 | 129,6 | 84 | 3 | 44 | 5 |
| Q61838 | Alpha-2-macroglobulin | 165,7 | 73 | 4 | 58 | 4 |
| Q91VB8 | Alpha globin 1 | 15,1 | 61 | 5 | ― | ― |
| Q00623 | Apolipoprotein A-I | 30,6 | 47 | 6 | 66 | 3 |
| P60710 | Actin, cytoplasmic 1 | 41,7 | 45 | 7 | 29 | 7 |
| P63260 | Actin, cytoplasmic 2 | 41,8 | 43 | 8 | 26 | 8 |
| A0A0R4J0I1 | MCG1051009 | 46,6 | 43 | 9 | 39 | 6 |
| P26039 | Talin-1 | 269,7 | 37 | 10 | 16 | 14 |
| A8DUK4 | Beta-globin | 15,7 | 32 | 11 | ― | ― |
| Q8VDD5 | Myosin-9 | 226,2 | 25 | 12 | 7 | 38 |
| P01027 | Complement C3 | 186,4 | 23 | 13 | 19 | 11 |
| Q91X72 | Hemopexin | 51,3 | 23 | 14 | 15 | 17 |
| P21614 | Vitamin D-binding protein | 53,6 | 22 | 15 | 19 | 12 |
| P01872 | Ig mu chain C region | 49,9 | 21 | 16 | 15 | 16 |
| Q9Z126 | Platelet factor 4 | 11,2 | 21 | 17 | 17 | 13 |
| Q99K47 | Fibrinogen, alpha polypeptide | 61,3 | 21 | 18 | 10 | 29 |
| A0A0R4J0X5 | Alpha-1-antitrypsin 1-3 | 45,8 | 19 | 19 | ― | ― |
| B7FAV1 | Filamin, alpha (Fragment) | 274,5 | 18 | 20 | 13 | 19 |
| P28665 | Murinoglobulin-1 | 165,2 | 17 | 21 | 13 | 20 |
| P17742 | Peptidyl-prolyl cis-trans isomerase A | 18,0 | 17 | 22 | 8 | 35 |
| Q9EQI5 | Chemokine (C-X-C motif) ligand 7, isoform CRA_b | 12,2 | 17 | 23 | 10 | 27 |
| P23953 | Carboxylesterase 1C | 61,0 | 16 | 24 | 11 | 22 |
| P20918 | Plasminogen | 90,7 | 16 | 25 | 11 | 24 |
| P09813 | Apolipoprotein A-II | 11,3 | 15 | 26 | 10 | 28 |
| Q8K0E8 | Fibrinogen beta chain | 54,7 | 14 | 27 | 7 | 39 |
| P05064 | Fructose-bisphosphate aldolase A | 39,3 | 14 | 28 | 10 | 30 |
| P22599 | Alpha-1-antitrypsin 1-2 (serpina) | 45,9 | 14 | 29 | 11 | 23 |
| O08677-2 | Isoform LMW of Kininogen-1 | 47,9 | 14 | 30 | 14 | 18 |
| P07758 | Alpha-1-antitrypsin 1-1 | 46,0 | 13 | 31 | ― | ― |
| P63101 | 14-3-3 protein zeta/delta | 27,8 | 13 | 32 | ― | ― |
| Q00897 | Alpha-1-antitrypsin 1-4 | 46,0 | 13 | 33 | 10 | 26 |
| P20029 | 78 kDa glucose-regulated protein | 72,4 | 11 | 34 | 3 | 71 |
| O88342 | WD repeat-containing protein 1 | 66,4 | 11 | 35 | 5 | 43 |
| P68373 | Tubulin alpha-1C chain | 49,9 | 11 | 36 | ― | ― |
| E9QPU1 | von Willebrand factor | 309,0 | 11 | 37 | 4 | 52 |
| P63017 | Heat shock cognate 71 kDa protein | 70,8 | 11 | 38 | 5 | 47 |
| G3X9T8 | Ceruloplasmin | 121,0 | 11 | 39 | 9 | 31 |
| P68368 | Tubulin alpha-4A chain | 49,9 | 10 | 40 | 7 | 37 |
| O35930 | Platelet glycoprotein Ib alpha chain | 80,0 | 10 | 41 | 6 | 40 |
| Q9DBD0 | Inhibitor of carbonic anhydrase | 76,7 | 10 | 42 | 9 | 32 |
| P62962 | Profilin-1 | 14,9 | 9 | 43 | ― | ― |
| P08905 | Lysozyme C-2 | 16,7 | 9 | 44 | 4 | 56 |
| P29699 | Alpha-2-HS-glycoprotein | 37,3 | 9 | 45 | 12 | 21 |
| Q6IRU2 | Tropomyosin alpha-4 chain | 28,5 | 9 | 46 | 4 | 54 |
| Q00898 | Alpha-1-antitrypsin 1-5 | 45,9 | 8 | 47 | 5 | 42 |
| D6RGQ0 | Complement factor H | 125,0 | 8 | 48 | ― | ― |
| P18760 | Cofilin-1 | 18,5 | 8 | 49 | 3 | 70 |
| Q7TQE2 | Zyx protein | 57,0 | 8 | 50 | 2 | 93 |
| P21107-2 | Isoform 2 of Tropomyosin alpha-3 chain | 29,0 | 8 | 51 | ― | ― |
| P06151 | L-lactate dehydrogenase A chain | 36,5 | 8 | 52 | 3 | 85 |
| Q01339 | Beta-2-glycoprotein 1 | 38,6 | 8 | 53 | ― | ― |
| P68510 | 14-3-3 protein eta | 28,2 | 8 | 54 | ― | ― |
| P17751 | Triosephosphate isomerase | 32,2 | 7 | 55 | 3 | 73 |
| Q8VCM7 | Fibrinogen gamma chain | 49,4 | 7 | 56 | 4 | 55 |
| P27773 | Protein disulfide-isomerase A3 | 56,6 | 7 | 57 | ― | ― |
| Q01853 | Transitional endoplasmic reticulum ATPase | 89,3 | 7 | 58 | ― | ― |
| P08226 | Apolipoprotein E | 35,8 | 7 | 59 | 4 | 53 |
| A2AQ07 | Tubulin beta-1 chain | 50,4 | 6 | 60 | 8 | 34 |
| Q64442 | Sorbitol dehydrogenase | 38,2 | 6 | 61 | ― | ― |
| P04186 | Complement factor B | 85,0 | 6 | 62 | 5 | 45 |
| Q60605 | Myosin light polypeptide 6 | 16,9 | 6 | 63 | ― | ― |
| Q64727 | Vinculin | 116,6 | 6 | 64 | 2 | 95 |
| A0A075B5P4 | Ig gamma-1 chain C region secreted form (Fragment) | 35,7 | 6 | 65 | 4 | 58 |
| A0A075B5P3 | Protein Ighg2b (Fragment) | 36,7 | 6 | 66 | 3 | 74 |
| A0A075B5V1 | Protein Ighv1-31 | 11,0 | 6 | 67 | 4 | 62 |
| A0A0J9YUQ8 | Gelsolin (Fragment) | 62,3 | 6 | 68 | ― | ― |
| P52480 | Pyruvate kinase PKM | 57,8 | 5 | 69 | 3 | 78 |
| Q02053 | Ubiquitin-like modifier-activating enzyme 1 | 117,7 | 5 | 70 | ― | ― |
| H3BJP2 | S-formylglutathione hydrolase (Fragment) | 27,1 | 5 | 71 | 3 | 87 |
| P40142 | Transketolase | 67,6 | 5 | 72 | ― | ― |
| P09411 | Phosphoglycerate kinase 1 | 44,5 | 5 | 73 | 2 | 94 |
| A0A075B5V0 | MCG114299 (Fragment) | 12,9 | 5 | 74 | ― | ― |
| Q9WVF5 | Epidermal growth factor receptor | 72,9 | 5 | 75 | ― | ― |
| Q91VW3 | SH3 domain-binding glutamic acid-rich-like protein 3 | 10,5 | 5 | 76 | 3 | 72 |
| P20065-2 | Isoform Short of Thymosin beta-4 | 5,0 | 5 | 77 | 6 | 41 |
| P06728 | Apolipoprotein A-IV | 45,0 | 5 | 78 | 9 | 33 |
| P61982 | 14-3-3 protein gamma | 28,3 | 5 | 79 | ― | ― |
| Q9ESB3 | Histidine-rich glycoprotein | 59,1 | 5 | 80 | 4 | 63 |
| A0A0A6YXN4 | Protein Ighv1-18 (Fragment) | 12,9 | 4 | 81 | ― | ― |
| A0A0R4J1N3 | Apolipoprotein C-III | 10,9 | 4 | 82 | 4 | 49 |
| Q9DBB9 | Carboxypeptidase N subunit 2 | 60,4 | 4 | 83 | 4 | 50 |
| Q9QWK4 | CD5 antigen-like | 38,8 | 4 | 84 | 4 | 60 |
| O54890 | Integrin beta-3 | 86,7 | 4 | 85 | 2 | 99 |
| P14152 | Malate dehydrogenase, cytoplasmic | 36,5 | 4 | 86 | ― | ― |
| O89020 | Afamin | 69,3 | 4 | 87 | 4 | 51 |
| Q61129 | Complement factor I | 67,2 | 4 | 88 | ― | ― |
| S4R2J8 | Bridging integrator 2 | 50,9 | 4 | 89 | ― | ― |
| P56480 | ATP synthase subunit beta, mitochondrial | 56,3 | 4 | 90 | ― | ― |
| Q9D6F9 | Tubulin beta-4A chain | 49,6 | 4 | 91 | 3 | 84 |
| E9Q453 | Tropomyosin alpha-1 chain | 28,5 | 4 | 92 | 2 | 98 |
| A0A075B5P2 | Protein Igkc (Fragment) | 11,9 | 4 | 93 | ― | ― |
| E9Q748 | Antileukoproteinase | 11,9 | 4 | 94 | ― | ― |
| Q7TMM9 | Tubulin beta-2A chain | 49,9 | 4 | 95 | ― | ― |
| Q61703 | Inter-alpha-trypsin inhibitor heavy chain H2 | 105,9 | 4 | 96 | 4 | 66 |
| P01654 | Ig kappa chain V-III region PC 2880/PC 1229 | 12,0 | 3 | 97 | ― | ― |
| Q8CG19 | Latent-transforming growth factor beta-binding protein 1 | 186,6 | 3 | 98 | ― | ― |
| P17182 | Alpha-enolase | 47,1 | 3 | 99 | ― | ― |
| A0A075B5N9 | Protein Igkv3-7 | 10,9 | 3 | 100 | ― | ― |
| P01942 | Hemoglobin subunit alpha | 15,1 | ― | ― | 23 | 9 |
| E9Q223 | Hemoglobin subunit beta-1 (Fragment) | 11,1 | ― | ― | 22 | 10 |
| Q00896 | Alpha-1-antitrypsin 1-3 | 45,8 | ― | ― | 16 | 15 |
| G3X8T9 | Serine (Or cysteine) peptidase inhibitor, clade A, member 3N, isoform CRA_a | 46,7 | ― | ― | 10 | 25 |
| A0A0A6YWP4 | Complement factor H (Fragment) | 94,2 | ― | ― | 8 | 36 |
| D3YXF4 | 14-3-3 protein zeta/delta (Fragment) | 5,1 | ― | ― | 5 | 44 |
| I7HJR3 | Beta-2-glycoprotein 1 (Fragment) | 16,8 | ― | ― | 5 | 46 |
| A0A0A6YYE7 | Protein Igkv4-57 (Fragment) | 12,7 | ― | ― | 4 | 48 |
| A0A0A6YWH7 | Antithrombin-III (Fragment) | 31,9 | ― | ― | 4 | 57 |
| P29788 | Vitronectin | 54,8 | ― | ― | 4 | 59 |
| P26262 | Plasma kallikrein | 71,3 | ― | ― | 4 | 61 |
| P01029 | Complement C4-B | 192,8 | ― | ― | 4 | 64 |
| Q19LI2 | Alpha-1B-glycoprotein | 56,5 | ― | ― | 4 | 65 |
| H7BX99 | Prothrombin | 70,2 | ― | ― | 4 | 67 |
| A0A0B4J1I9 | Protein Igkv4-55 (Fragment) | 12,7 | ― | ― | 3 | 68 |
| Q07456 | Protein AMBP | 39,0 | ― | ― | 3 | 69 |
| Q99PT1 | Rho GDP-dissociation inhibitor 1 | 23,4 | ― | ― | 3 | 75 |
| O55222 | Integrin-linked protein kinase | 51,3 | ― | ― | 3 | 76 |
| O70400 | PDZ and LIM domain protein 1 | 35,8 | ― | ― | 3 | 77 |
| Q9QUM0 | Integrin alpha-IIb | 112,6 | ― | ― | 3 | 79 |
| Q91XL1 | Leucine-rich HEV glycoprotein | 37,4 | ― | ― | 3 | 80 |
| A2A997 | Complement component C8 alpha chain | 65,4 | ― | ― | 3 | 81 |
| P01660 | Ig kappa chain V-III region PC 3741/TEPC 111 | 12,1 | ― | ― | 3 | 82 |
| D3Z6I8 | Tropomyosin alpha-3 chain | 28,7 | ― | ― | 3 | 83 |
| P42703-2 | Isoform 2 of Leukemia inhibitory factor receptor | 81,2 | ― | ― | 3 | 86 |
| P03987-2 | Isoform 2 of Ig gamma-3 chain C region | 36,2 | ― | ― | 3 | 88 |
| P01592 | Immunoglobulin J chain | 18,0 | ― | ― | 2 | 89 |
| Q8BH35-2 | Isoform 2 of Complement component C8 beta chain | 58,4 | ― | ― | 2 | 90 |
| A0A0A6YY53 | Protein Ighg2c (Fragment) | 36,5 | ― | ― | 2 | 91 |
| Q9DAC2 | Complement component 8, gamma subunit, isoform CRA_b | 18,9 | ― | ― | 2 | 92 |
| Q8K1B8 | Fermitin family homolog 3 | 75,6 | ― | ― | 2 | 96 |
| O88947 | Coagulation factor X | 54,0 | ― | ― | 2 | 97 |
| Q07968 | Coagulation factor XIII B chain | 76,1 | ― | ― | 2 | 100 |
